# Sustainable Graphene-Reinforced Geopolymer Composites Derived from Recycled Volcanic Ash: Matrix Optimization, Characterization, and Photoluminescent Proof-of-Concept

**DOI:** 10.3390/polym18192322

**Published:** 2026-09-23

**Authors:** Luigi Madeo, Carlo Poselle Bonaventura, Paolino Caputo, Anastasia Macario, Sebastiano Candamano, Peppino Sapia, Cesare Oliviero Rossi, Alfonso Policicchio, Pierantonio De Luca

**Affiliations:** 1 Department of Environmental Engineering (DIAm), University of Calabria, 87036 Rende, CS, Italy; anastasia.macario@unical.it; 2 Department of Physics, University of Calabria, 87036 Rende, CS, Italy; carlo.poselle@unical.it (C.P.B.); alfonso.policicchio@fis.unical.it (A.P.); 3 Department of Chemistry and Chemical Technologies (CTC), University of Calabria, 87036 Rende, CS, Italy; paolino.caputo@unical.it (P.C.); cesare.oliviero@unical.it (C.O.R.); 4 Department of Mechanical, Energy and Management Engineering (DIMEG), University of Calabria, 87036 Rende, CS, Italy; sebastiano.candamano@unical.it; 5 Department of Mathematics and Computer Science (DEMACS), University of Calabria, 87036 Rende, CS, Italy; peppino.sapia@unical.it

**Keywords:** geopolymers, etna volcanic ash, circular economy, waste valorization, graphene nanoparticles, photoluminescence, hybrid composites, epoxy resin, smart materials

## Abstract

This study presents the development of sustainable and multifunctional geopolymer composites produced from recycled volcanic ash collected from Mount Etna (Italy), used as an aluminosilicate precursor for the fabrication of environmentally friendly materials. Geopolymerization was activated using a 6 M sodium hydroxide (NaOH) solution. The geopolymer matrix was optimized by investigating three liquid-to-solid (L/S) ratios (0.2, 0.3, and 0.4) in order to identify the most suitable formulation in terms of microstructure and porosity. SEM, EDS, BET, particle size distribution (PSD), and X-ray diffraction (XRD) analyses identified an L/S ratio of 0.3 as the optimal formulation. Based on this optimized matrix, graphene nanoparticles were incorporated at concentrations of 0.1, 0.2, and 0.5 wt.% to evaluate their influence on the microstructural, thermal, mechanical, and durability-related properties of the composites. The resulting materials were characterized by SEM, EDS, XRD, differential scanning calorimetry (DSC), mechanical testing, and water absorption measurements. Finally, hybrid prototypes consisting of geopolymer bars coated with a transparent epoxy resin containing photoluminescent powder were fabricated. Their photoluminescent performance was qualitatively evaluated following direct solar irradiation by monitoring the persistence of light emission over time. The results demonstrate the feasibility of integrating a geopolymer matrix derived from volcanic waste, graphene nanoparticles, and a photoluminescent coating into a single sustainable and multifunctional material. Although this work represents a preliminary proof-of-concept study, it provides a promising foundation for the development of smart geopolymer-based materials for applications in civil infrastructure, passive safety systems, and guidance devices operating under low-light conditions.

## 1. Introduction

Geopolymers, or aluminosilicate-based inorganic polymers, are emerging as one of the most promising classes of materials within the framework of the ecological transition applied to the construction, interior architecture, and urban infrastructure sectors [1,2,3]. Owing to their significantly lower carbon footprint compared with conventional cementitious binders, they represent a sustainable alternative characterized by several attractive properties, including rapid hardening kinetics, high thermal stability, and excellent chemical durability, even under aggressive environmental conditions. These features make geopolymers particularly suitable matrices for the development of environmentally friendly functional components and products [4,5,6,7].

The geopolymerization process involves the dissolution of silica (SiO_2_)- and alumina (Al_2_O_3_)-rich solid precursors in a highly alkaline solution, followed by the condensation of the dissolved species into a chemically stable three-dimensional amorphous or semi-crystalline aluminosilicate network [8,9,10,11]. Although the scientific literature has extensively focused on secondary industrial precursors such as fly ash and blast furnace slag, current research is increasingly oriented toward the principles of the circular economy and waste valorization [12,13,14]. The main objective is to identify low-cost natural wastes or geological by-products that can be regenerated and strategically reused in new engineering and industrial applications with high added value, while simultaneously reducing the environmental impact associated with their disposal [15,16].

Within this context, volcanic regions offer enormous yet still largely underexploited potential. Mount Etna (Italy), one of the most active volcanoes in the world, periodically produces large quantities of volcanic ash and lava dust during its eruptive events [17,18]. This material accumulates extensively in urban areas and along surrounding road networks, creating significant environmental management challenges and imposing considerable removal costs on local authorities [19]. From a chemical and structural perspective, however, volcanic ash represents a naturally occurring aluminosilicate resource that is widely available, essentially cost-free, and characterized by latent chemical reactivity, making it an excellent precursor for alkali-activated material synthesis [20].

The primary motivation of the present work lies in the opportunity to combine the sustainability of the raw materials with the simplicity of the production process, which is a key requirement for the effective transfer of research outcomes from laboratory-scale investigations to real-world applications. Advanced materials are often produced through energy-intensive preliminary treatments, such as calcination, or complex chemical synthesis routes that compromise their environmental benefits [21]. In contrast, this study adopts a low-energy and low-cost approach by implementing a simple and scalable processing protocol based on the direct cold mixing of untreated Etna volcanic ash with a 6 M sodium hydroxide (NaOH) solution, without any thermal pre-treatment of the precursor. To optimize the properties of the geopolymeric system, particular attention was devoted to the control of porosity and microstructural development, both of which are strongly influenced by the liquid-to-solid (L/S) ratio of the mixture [22,23]. In parallel with the optimization of the L/S ratio, recent advances in materials science have highlighted the incorporation of nanomaterials as an effective strategy for tailoring the internal microstructure and reducing the microscopic voids typically found in geopolymer matrices [24]. In this regard, the incorporation of low weight fractions of graphene nano-powder represents a particularly promising approach [25]. Owing to its exceptionally high specific surface area and unique structural characteristics, graphene can be incorporated into the aluminosilicate network, acting as both a nucleation agent and a microstructural modifier, with the potential to reduce diffuse microcracking while preserving the overall economic and environmental sustainability of the geopolymer formulation [26]. In parallel with the development of sustainable materials, there is an increasing demand for multifunctional materials capable of providing additional functionalities without the need for external power sources. In this context, the combination of geopolymers with organic polymers offers promising opportunities for the development of hybrid composites [27,28]. The use of a transparent epoxy resin represents an effective solution owing to its ease of cold casting, low cost, and excellent chemical stability [29,30]. Moreover, the transparent nature of the epoxy phase enables the incorporation and stable immobilization of photoluminescent particles (phosphors) within the composite system. This approach protects the photoluminescent additive from the highly alkaline environment of the geopolymer matrix, which could otherwise lead to the degradation of its optical properties, while simultaneously ensuring excellent particle retention and a homogeneous distribution over the surface of the manufactured component [31,32].

The resulting hybrid composites exhibit a remarkable ability to absorb and store light energy from both artificial light sources, such as UV lamps, and natural solar irradiation, followed by prolonged light emission under dark conditions [33,34,35]. This photoluminescent behavior opens new opportunities for the development of smart materials for nighttime safety and energy-saving applications [36,37]. Potential applications include passive road safety elements and urban furniture capable of improving visibility in the absence of public lighting, architectural components and interior finishing materials for public and private buildings, as well as self-illuminating pedestrian walkways and bicycle paths [38,39,40]. Accordingly, the present work aims to investigate the entire development process of these novel hybrid materials, from the optimization of the liquid-to-solid (L/S) ratio of the Etna volcanic ash-based geopolymer matrix, evaluated by SEM, EDS, XRD, and BET/PSD porosity analyses, to the assessment of the effect of graphene nanoparticle incorporation on the microstructure, and finally to the validation of the optical performance of the epoxy-based photoluminescent coating. Overall, this study proposes an integrated, low-cost, and circular strategy for the development of sustainable and multifunctional materials with potential applications in next-generation civil engineering and smart infrastructure systems.

## 2. Materials and Methods

This section describes the raw materials used and the experimental protocols followed for the synthesis of volcanic ash-based geopolymers, their structural optimization through graphene nanoparticle addition, the preparation of photoluminescent hybrid prototypes, and the related physicochemical characterization techniques.

### 2.1. Raw Materials

- Aluminosilicate precursor: Volcanic ash collected from Mount Etna (Sicily, Italy). The raw material was previously subjected to mechanical grinding to obtain a fine and homogeneous powder. Before each use, the volcanic ash powder was thermally pre-treated in a static oven at 100 °C for 12 h to completely remove residual moisture.
- Alkaline activator: A 6 M aqueous sodium hydroxide (NaOH) solution, prepared by dissolving analytical-grade sodium hydroxide pellets (Carlo Erba Reagents S.r.l., Cornaredo, Italy) in distilled water.
- Nano-additive: Graphene nanoplatelets (GH) (PlasmaChem GmbH, Berlin, Germany; purity: 99.0%).
- Organic binder: Transparent epoxy resin (Resina Epossidica 5000 C. 4K, Component A + Component B, Colorpoint S.r.l., Bellizzi (SA), Italy).
- Functional additive: Commercial photoluminescent phosphor powder (manufacturer not specified by supplier), shipped from Guangzhou, China. Further information on the nature of the photoluminescent material is reported in a previous work [27].

### 2.2. Synthesis of Geopolymer Composites

The synthesis procedure was divided into two consecutive stages: (i) optimization of the liquid-to-solid (L/S) ratio and (ii) subsequent graphene addition into the optimized geopolymer matrix.

(i) Phase 1—L/S ratio optimization:

A 6 M NaOH activating solution was used for geopolymer synthesis after cooling and reaching room temperature before use. Three formulations were prepared by keeping the volcanic ash content constant (30 g) and varying the amount of alkaline activating solution to obtain liquid-to-solid ratios (L/S) of 0.2, 0.3, and 0.4, corresponding to 6 g, 10 g, and 13 g of activating solution, respectively. The mixtures were manually blended using a laboratory spatula for approximately 3 min until a homogeneous geopolymer paste was obtained. The 0.2 L/S ratio resulted in a very dry and stiff paste, difficult to cast; the 0.3 L/S ratio showed good workability and homogeneous casting; the 0.4 L/S ratio produced a very fluid paste, which was not ideal for mold filling and shape stability.

(ii) Phase 2—Graphene addition:

After selecting the L/S ratio of 0.3 as the optimal formulation (Geop 2 specimen), graphene nanoplatelets were incorporated into the optimized matrix at three different weight percentages: 0.1 wt% (0.030 g), 0.2 wt% (0.060 g), and 0.5 wt% (0.150 g), calculated with respect to the volcanic ash mass (30 g). The graphene powder was first dry-mixed with the volcanic ash to ensure uniform dispersion. Subsequently, 10 g of alkaline activating solution was added, and the mixtures were manually homogenized to obtain the three graphene-modified geopolymer formulations.

All geopolymer pastes obtained from Phase 1 and Phase 2 were cast into suitable silicone molds resistant to high temperatures. The specimens were then subjected to thermal curing in a static oven (VWR DRY-Line DL 53, Langenfeld, Germany) previously heated to approximately 110 °C for 24 h to promote the geopolymerization reaction. Following the curing process, the oven was turned off and the specimens were allowed to cool slowly inside the chamber before being removed from the molds. The designation of the geopolymer formulations and their respective compositions are reported in Table 1, whereas the prepared specimens obtained after demolding are presented in Figure 1.

### 2.3. Preparation of the Hybrid Photoluminescent Coating

For the evaluation of photoluminescent properties, the optimized geopolymer specimens were produced in the form of laboratory-scale bars and subsequently coated on the upper surface with a hybrid photoluminescent coating. The coating formulation was kept constant for all samples and was prepared using 7.5 g of transparent epoxy resin (Component A), 5.0 g of hardener (Component B), and 1.0 g of commercial photoluminescent powder. The materials used for the preparation of the photoluminescent coating are shown in Figure 2.

The components were manually mixed using a spatula (Emi style, RGM, Maniago, Italy) for approximately 3 min until a homogeneous mixture, free of visible agglomerates, was obtained, with a viscosity suitable for the subsequent casting process. The photoluminescent mixture was then uniformly applied onto the upper surface of the previously prepared geopolymer specimens. Immediately after casting, the silicone molds containing the specimens were gently tapped to promote the release of any entrapped air bubbles and minimize the formation of defects during the curing stage. The curing process was carried out at room temperature for 24 h, a period considered sufficient to ensure complete hardening of the epoxy coating and to allow safe demolding of the prototypes without the occurrence of cracks, detachment phenomena, or surface defects. The resulting photoluminescent geopolymer specimens after removal from the silicone molds are shown in Figure 3.

### 2.4. Characterization Techniques

#### 2.4.1. Microstructural and Elemental Analysis (SEM/EDS)

The morphology of the reaction products, matrix homogeneity, and surface microstructural features of the geopolymeric specimens were investigated by scanning electron microscopy (SEM) using a Zeiss Crossbeam 350 Gemini microscope (Carl Zeiss Microscopy GmbH, Oberkochen, Germany). Prior to SEM analysis, all fractured specimens were subjected to vacuum deposition of an ultrathin conductive coating using a sputter coater to improve electrical conductivity and minimize charging effects during imaging. The SEM system was coupled with an energy-dispersive X-ray spectroscopy (EDS) ZEISS Gemini SEM Crossbeam 350 (Carl Zeiss Microscopy GmbH, Oberkochen, Germany) detector to perform elemental microanalysis and semi-quantitative chemical characterization of the geopolymer matrix. This combined approach was employed to evaluate the development of the geopolymeric structure, the characteristics of the reaction products, and the distribution of graphene nanoplatelets within the modified matrices.

#### 2.4.2. X-Ray Diffraction (XRD)

X-ray diffraction (XRD) analysis was performed to identify the crystalline phases remaining from the volcanic precursor and to investigate the structural changes associated with the geopolymerization process. In addition, XRD patterns were analyzed to evaluate possible modifications related to the incorporation of graphene nanoplatelets within the geopolymer matrix. The analyses were carried out using a Rigaku MiniFlex 600 X-ray diffractometer (Rigaku Corporation, Tokyo, Japan) equipped with Cu Kα radiation, operated at 20 mA and 40 kV. Powdered samples were scanned over a 2θ range of 5–50° with a step size of 0.02° and a scanning rate of 1°/min.

#### 2.4.3. Physisorption and Pore Size Distribution (BET/PSD)

Nitrogen adsorption–desorption measurements were carried out at 77 K using a Micromeritics ASAP 2460 (Micromeritics Instrument Corporation, Norcross, GA, USA) analyzer to investigate the textural properties of the samples. Before each analysis, the materials were evacuated at 353 K for 24 h to remove adsorbed species. The specific surface area (SSA) was obtained from the Brunauer–Emmett–Teller equation within the relative pressure interval (P/P°= 0.05–0.30), following the Rouquerol consistency criteria [41]. Pore volume values were estimated from the desorption branch of the isotherms by means of the Barrett–Joyner–Halenda method [42], considering the (P/P°) range between 0.40 and 0.99 and adopting the Harkins–Jura thickness equation [43].

#### 2.4.4. Thermal Analysis DSC

DSC analyses were performed to investigate the thermal behavior of the geopolymer matrices and to evaluate possible modifications induced by graphene nanoplatelet incorporation. The measurements were carried out using a DSC 131 Differential Scanning Calorimeter (SETARAM Instrumentation, Caluire, France). For each test, approximately 36.0 mg (0.0360 g) of finely ground sample was placed in an aluminum crucible. The thermal program consisted of an initial isothermal step at 30 °C for 10 min, followed by a linear heating ramp from 30 °C to 500 °C at a rate of 20 °C/min under a continuous nitrogen (N_2_) flow to provide an inert atmosphere. The obtained thermograms were analyzed to compare the thermal response of the reference geopolymer matrix and graphene-modified formulations.

#### 2.4.5. Water Absorption Test

The water absorption capacity by total immersion was evaluated on cured geopolymer bars before the application of the photoluminescent resin coating, in order to determine the water-accessible porosity and the material behavior under humid conditions.

Prior to the immersion test, the specimens were subjected to a drying procedure aimed at removing residual moisture contained within the geopolymer matrix. For this purpose, a vacuum desiccator containing silica gel previously regenerated by thermal treatment in an oven at 100 °C for 2 h was used. The specimens were maintained under vacuum conditions inside the desiccator for 24 h until constant mass was achieved.

After the drying process, the dry mass of each specimen (m_d_) was measured using an analytical balance (OHAUS Analytical Plus AP250D, Greifensee, Switzerland) with a precision of ±0.00001 g.

Subsequently, each specimen was completely immersed in distilled water at room temperature for 24 h. After immersion, the samples were removed from the water, gently blotted with absorbent paper to eliminate excess surface water while avoiding the removal of water absorbed within the pore network, and immediately weighed to determine the saturated mass (m_s_).

The water absorption capacity was calculated as the percentage increase in mass relative to the dry mass according to the following equation:

$$WA\left[\%\right]=\frac{m_{s}-m_{d}}{m_{d}}$$

where:

WA = water absorption (%);

m_s_ = dry mass of the specimen after vacuum drying (g);

m_d_ = saturated mass of the specimen after 24 h immersion in water (g).

The preliminary drying procedure was carried out to ensure uniform initial conditions among the specimens and to improve the accuracy of water absorption measurements by removing any residual moisture present within the porous structure of the material.

#### 2.4.6. Mechanical Testing

The mechanical behavior of the geopolymer specimens was preliminarily investigated through comparative compression tests using a digital durometer (VanderKamp VK200, VanKel Industries, Inc., Edison, NJ, USA), which records the maximum load applied at specimen failure, expressed in kiloponds (kp), with a maximum measuring capacity of 35 kp. The aim of these tests was not to determine standardized compressive strength values according to international testing procedures, but rather to provide a preliminary comparative assessment of the mechanical response of the different formulations and to identify possible variations induced by graphene nanoplatelet incorporation into the reference geopolymer matrix.

Since the expected mechanical resistance of standard-sized geopolymer specimens exceeded the measurement range of the available instrument, cubic specimens with dimensions of 1 × 1 × 1 cm^3^ were specifically prepared for each formulation. The reduced specimen size allowed the mechanical response to be evaluated within the operating range of the equipment while maintaining identical testing conditions for all samples.

The compression tests were performed under the same experimental conditions for all formulations, enabling a comparative evaluation of the influence of graphene addition on the mechanical behavior of the geopolymer matrix. The load values recorded by the instrument were converted from kiloponds (kp) to kilonewtons (kN) and are reported in the Results section for comparative evaluation among the investigated formulations.

The obtained values should be interpreted as preliminary mechanical indicators of the effect of graphene incorporation rather than standardized compressive strength measurements determined according to international testing standards.

The experimental setup and the mechanical testing procedure are shown in Figure 4.

#### 2.4.7. Preliminary Qualitative Evaluation of the Photoluminescent Behavior

A preliminary qualitative evaluation was performed to investigate the photoluminescent behavior of the developed geopolymer-based composites and to verify the feasibility of integrating a photoluminescent epoxy coating onto the geopolymer substrates. Particular attention was devoted to assessing whether the presence of graphene nanoplatelets at different concentrations within the geopolymer matrix could produce observable differences in the persistence of the photoluminescent emission (afterglow) after natural solar activation.

The purpose of this investigation was to provide a comparative visual assessment of the developed formulations rather than a quantitative optical characterization. Therefore, no instrumental measurements of luminance, luminous intensity, quantum efficiency, or emission spectra were performed. The evaluation was exclusively based on visual observation of the afterglow persistence and photographic documentation collected over time.

All geopolymer formulations (Geop 2, Geop 4, Geop 5, and Geop 6) were evaluated under identical experimental conditions. Prior to the photoluminescence assessment, all specimens were simultaneously exposed to direct natural sunlight for 15 min during the period of maximum daily solar irradiance (approximately 12:00 p.m.) to provide comparable activation conditions for the photoluminescent phosphors contained in the epoxy coating. The environmental conditions reported in Table 2 were collected from publicly available meteorological sources and official weather databases referring to the experimental location during the solar activation period.

Immediately after the activation period, the specimens were transferred into a completely dark environment. The persistence of the visible afterglow was monitored through direct visual observation and documented by photographic acquisition at predefined time intervals until the emitted light was no longer visually distinguishable under the adopted observation conditions. The photographic documentation was used solely for qualitative comparison among the investigated geopolymer formulations, allowing the identification of possible differences in the persistence of photoluminescent emission associated with the incorporation of graphene nanoplatelets. No digital image processing or quantitative optical analysis was performed, as the objective of this preliminary investigation was to demonstrate the feasibility of the proposed photoluminescent geopolymer prototypes and to identify potential trends requiring further quantitative investigation.

The results of this qualitative assessment are presented and discussed in the Results section together with the corresponding photographic documentation.

## 3. Results and Discussion

The results obtained in this study are presented following the sequential development of the experimental approach. First, the optimal liquid-to-solid (L/S) ratio was identified by evaluating the microstructural, compositional, textural, and mineralogical features of the geopolymer matrices through SEM, EDS, BET, and XRD analyses. This preliminary optimization step allowed the selection of the most suitable reference formulation for subsequent graphene incorporation.

Based on the optimized geopolymer composition, graphene-modified formulations were then investigated by introducing different graphene nanoplatelet contents. The resulting composites were characterized through a combined analytical approach including SEM, EDS, BET, XRD, DSC, preliminary mechanical evaluation, and water absorption measurements, with the aim of assessing possible modifications induced by graphene addition on the structural, thermal, and functional properties of the geopolymer matrix.

Finally, the photoluminescent behavior of the developed prototypes was evaluated after application of the luminescent epoxy coating. A qualitative time-dependent visual assessment was performed to investigate the persistence of the afterglow emission under natural solar activation conditions and to provide a preliminary evaluation of the potential of the proposed geopolymer-based multifunctional composites.

### 3.1. Optimization of the Geopolymer Matrix Through L/S Ratio Evaluation

#### 3.1.1. Textural Characterization by SEM and EDS Microanalysis

The influence of the liquid-to-solid (L/S) ratio on the microstructural development and elemental composition of the geopolymer matrices was investigated through SEM imaging coupled with EDS microanalysis. This combined approach was used to compare the morphology of the different formulations and to evaluate compositional variations associated with the different amounts of alkaline activating solution. The SEM micrographs and corresponding EDS spectra of the Geop 1, Geop 2, and Geop 3 samples are reported in Figure 5.

The EDS analyses revealed variations in the elemental composition of the geopolymer matrices as a function of the liquid-to-solid (L/S) ratio. An increase in sodium content was observed with increasing L/S ratio, consistent with the higher amount of NaOH activating solution introduced during the preparation of the corresponding formulations. In addition to sodium, all samples exhibited the typical elemental signals associated with alkali-activated aluminosilicate systems, including Si, Al, and O, which are consistent with the development of an aluminosilicate gel phase. The detection of calcium suggests the possible contribution of Ca-containing reaction products, particularly due to the intrinsic composition of the volcanic precursor. Other elements, such as Mg, K, Fe, and Ti, can be attributed to the original mineralogical composition of the volcanic ash and to residual phases not fully involved in the reaction process.

SEM observations highlighted the influence of the L/S ratio on the resulting microstructural features. The Geop 1 formulation, prepared with the lowest amount of activating solution, exhibited a relatively compact morphology characterized by limited porosity and a more continuous structure, suggesting reduced dissolution and reaction mobility due to the lower liquid availability. Conversely, the Geop 3 formulation showed a more open and heterogeneous morphology, with larger pores and irregular regions, likely associated with the higher amount of liquid phase introduced during preparation.

The intermediate formulation (Geop 2, L/S = 0.3) exhibited a more balanced microstructure, characterized by a homogeneous distribution of reacted and unreacted regions, limited macroporosity, and a more continuous geopolymeric network compared with the other investigated formulations. These features suggest that this composition provides a favorable balance between precursor dissolution, ionic mobility, and formation of the geopolymeric structure.

Overall, the combined SEM–EDS results indicate that the L/S ratio plays a key role in controlling the microstructural development of the geopolymer matrix. Among the investigated formulations, Geop 2 was selected as the optimal reference composition because it exhibited the most favorable morphological characteristics, representing a suitable compromise between structural compactness and reaction development for subsequent graphene modification.

#### 3.1.2. XRD Analysis to Investigate Potential Changes in Mineralogical Characteristics

X-ray diffraction (XRD) analysis was performed to investigate the mineralogical characteristics of the geopolymer formulations and to evaluate possible structural changes induced by the variation of the liquid-to-solid (L/S) ratio. The comparison of the diffraction patterns was performed to qualitatively assess the crystalline phases retained from the volcanic precursor and evaluate differences among the geopolymer matrices obtained under different activation conditions. The XRD patterns of the three geopolymer formulations synthesized from Etna volcanic ash (Geop 1: L/S = 0.2; Geop 2: L/S = 0.3; Geop 3: L/S = 0.4) are shown in Figure 6.

The comparative analysis of the XRD patterns of Geop 1, Geop 2, and Geop 3 samples allowed the qualitative evaluation of the main mineralogical characteristics and possible structural variations associated with changes in the liquid-to-solid (L/S) ratio. All diffraction patterns exhibit a broad increase of the baseline in the range of approximately 2θ = 20–38°, which can be attributed to the presence of a predominantly amorphous component. This feature is commonly observed in aluminosilicate-based geopolymeric systems and is consistent with the development of a disordered gel phase resulting from the partial dissolution of the reactive fraction of volcanic ash during alkaline activation.

Superimposed on the amorphous contribution, several crystalline reflections are observed, which are compatible with residual mineral phases originating from the Etna volcanic precursor. The main observed reflections are compatible with plagioclase- and pyroxene-related phases, with possible contributions from other residual minerals inherited from the volcanic precursor. These crystalline components may remain embedded within the geopolymeric matrix as unreacted fractions or mineral filler phases. The comparison among the three diffraction patterns reveals limited variations in the overall mineralogical profile, indicating that changes in the L/S ratio do not modify in evident mode, the main crystalline phases of the system but may influence the development of the geopolymeric matrix and the distribution of reaction products.

The Geop 1 formulation (L/S = 0.2), characterized by a lower amount of activating solution, may experience reduced liquid availability for precursor dissolution and ionic transport during the initial stages of reaction. Conversely, the Geop 3 formulation (L/S = 0.4) contains a higher liquid fraction, which may affect the effective concentration of alkaline species and the subsequent evolution of the microstructure during thermal curing.

Among the investigated formulations, Geop 2 (L/S = 0.3) was selected as the reference matrix for the subsequent graphene nanoparticle incorporation studies. This choice was based on the combined interpretation of SEM, EDS, BET, and XRD results, which indicated a favorable balance between geopolymeric structure development, matrix compactness, and porosity control.

#### 3.1.3. Textural Characterization (PSD)

The textural properties of Geop 1, Geop 2, and Geop 3 were investigated by N_2_ adsorption–desorption measurements at 77 K.

As shown in Figure 7, all samples exhibit limited nitrogen uptake at low relative pressures, followed by a gradual increase and a pronounced upturn at P/P° > 0.8. The separation between the adsorption and desorption branches in the intermediate-to-high relative-pressure region indicates capillary condensation within the accessible porous structure.

According to the IUPAC classification, the profiles can be described as type IV(a)-like isotherms with an H3-type hysteresis loop, generally associated with non-rigid aggregates and slit-shaped or interparticle pores. The absence of a marked uptake at P/P° < 0.1 suggests a negligible contribution from microporosity. Therefore, the accessible porosity is mainly related to mesopores, larger textural pores, and interparticle voids.

Overall, nitrogen uptake progressively increases from GEOP_1 to GEOP_3, in agreement with the surface-area and pore-volume data reported in Table 3. GEOP_3 exhibits the most developed accessible pore network, whereas GEOP_1 shows the most compact structure.

### 3.2. Effect of Graphene Content on the Characterization and Properties of Geopolymer Composites

Once the reference matrix Geop 2 (L/S ratio = 0.3) was selected as the most suitable formulation for further investigation, different amounts of graphene nanoplatelets (GH) were incorporated into this geopolymeric system to evaluate the effect of the nanofiller addition. Specifically, starting from the Geop 2 matrix, composite formulations containing 0.1 wt% (Geop 4), 0.2 wt% (Geop 5), and 0.5 wt% (Geop 6) of graphene were prepared.

The aim of this investigation was to evaluate how graphene incorporation could modify and potentially improve the main characteristics of the geopolymeric matrix, affecting its microstructural development, pore distribution, and overall homogeneity.

#### 3.2.1. Textural Characterization by SEM and EDS Microanalysis

The effect of graphene nanoplatelet incorporation on the geopolymer matrix was investigated through SEM imaging coupled with EDS microanalysis. This combined approach was used to evaluate possible morphological modifications, matrix homogeneity, and variations in elemental composition induced by graphene addition while maintaining the optimized L/S ratio of the reference formulation. The SEM micrographs and corresponding EDS spectra of the graphene-modified geopolymer samples (Geop 4, Geop 5, and Geop 6) are presented in Figure 8.

The SEM images of the graphene-containing geopolymer samples show a microstructural appearance that maintains the general features observed for the reference geopolymer matrix, while exhibiting some morphological differences associated with the incorporation of the nanomaterial. The introduction of lamellar carbon-based nanomaterials such as graphene nanoplatelets has been reported to potentially influence the development of alkali-activated matrices by modifying the arrangement of reaction products and the organization of the microstructural domains. The obtained micrographs reveal no evident increase in the presence of large voids or macroporous regions after graphene incorporation. Instead, the graphene-modified formulations exhibit a more complex and heterogeneous surface morphology compared with the unmodified geopolymer, which may be associated with interactions between the nanoplatelets and the developing geopolymeric network. The observed surface irregularities do not necessarily indicate a lower matrix compactness, as SEM analysis provides mainly local morphological information and does not allow a direct quantitative evaluation of the overall porosity. A more detailed assessment of the effect of graphene addition on the porous structure is provided by the BET analysis discussed in the following section. The decrease in specific surface area and pore volume observed with increasing graphene content suggests a modification of the accessible pore network, supporting the hypothesis that graphene incorporation may influence the packing arrangement and microstructural organization of the geopolymer matrix. Overall, SEM observations indicate that graphene addition alters the surface morphology of the geopolymer without producing evident detrimental effects on matrix integrity. The combined interpretation of SEM and BET results provides a more comprehensive evaluation of the influence of graphene nanoplatelets on the structural characteristics of the developed composites.

The observed reduction in specific surface area and pore volume after graphene incorporation suggests a modification of the accessible pore network and a possible increase in matrix compactness. Such microstructural changes may contribute to improved mechanical behavior by reducing critical defects and limiting stress concentration sites within the geopolymer structure. However, the effective influence of graphene addition on mechanical performance is discussed separately based on the results of the comparative mechanical tests.

#### 3.2.2. X-Ray Diffraction Analysis of Graphene-Containing Geopolymer Samples

The comparative analysis of the diffraction patterns (Figure 9) was performed to qualitatively evaluate the influence of graphene addition on the overall mineralogical profile of the geopolymer matrix.

The comparative analysis of the XRD patterns of graphene-modified samples (Geop 4, Geop 5, and Geop 6) and their comparison with the reference geopolymer matrix (Geop 2) allowed the evaluation of the effect of graphene incorporation on the mineralogical and structural characteristics of the developed system.

- Matrix stability and graphene incorporation:

All graphene-containing formulations exhibit diffraction patterns comparable to those of the Geop 2 reference matrix, maintaining the broad amorphous halo in the range of 2θ ≈ 20–38° and the main crystalline reflections compatible with residual mineral phases from the volcanic precursor, mainly attributed to plagioclase- and pyroxene-related phases. This behavior indicates that graphene addition at the investigated concentrations does not promote the formation of detectable new crystalline phases and does not modify the overall mineralogical profile of the geopolymeric matrix.

- Effect of low graphene concentrations (0.1% and 0.2 wt%):

Geop 4 and Geop 5 samples exhibit highly comparable XRD patterns, closely resembling the reference formulation. The absence of diffraction changes suggests that the incorporation of low graphene contents does not substantially alter the structural characteristics of the geopolymer matrix and remains compatible with the developed system. A slight variation can be observed around 2θ ≈ 26°; however, this contribution cannot be unambiguously assigned to graphene due to possible overlap with diffraction signals originating from residual volcanic mineral phases.

- Effect of increased graphene content (0.5 wt%):

The Geop 6 formulation shows a more evident modification of the overall diffraction profile compared with the lower graphene-containing samples. This behavior may be related to the higher amount of nanofiller incorporated into the matrix, although no additional crystalline phases can be clearly identified. The result suggests that higher graphene concentrations can influence the diffraction response of the composite without altering the mineralogical nature of the geopolymeric structure.

Overall, the XRD results indicate that graphene incorporation at the investigated concentrations preserves the main structural characteristics of the reference geopolymer matrix. The formulation containing 0.5 wt% graphene exhibits the most pronounced influence on the diffraction profile, suggesting a more pronounced influence of the nanofiller on the diffraction response of the composite, which will be further discussed in relation to the microstructural, thermal, and mechanical results.

#### 3.2.3. Textural Characterization (PSD)

The N_2_ adsorption–desorption isotherms of GEOP_4, GEOP_5, and GEOP_6 are shown in Figure 10.

All samples display very limited nitrogen uptake at low and intermediate relative pressures, followed by a more evident increase near saturation. The presence of hysteresis confirms the occurrence of capillary condensation within mesoporous and interparticle domains.

The isotherms may also be classified as type IV(a)-like with an H3-type hysteresis contribution. Their shape, together with the absence of a clear saturation plateau, is consistent with slit-shaped pores and voids formed between geopolymer aggregates. The low uptake at P/P° < 0.1 further indicates that microporosity is negligible. The textural parameters determined by BET analysis and reported in Table 4 confirm the differences observed from the nitrogen adsorption–desorption isotherms, highlighting the different accessibility of the porous structure among the investigated samples.

Among these samples, GEOP_4 shows the highest nitrogen uptake and the most developed accessible porosity, whereas GEOP_5 and GEOP_6 exhibit similarly low textural properties. As reported in Table 4, the second group is characterized by lower surface areas and pore volumes than GEOP_1–GEOP_3, indicating denser and less N_2_-accessible material.

The decrease in textural parameters does not necessarily imply a deterioration of the material performance, but rather highlights a microstructural modification induced by the presence of graphene. The graphene nanoparticles may act as a filling agent (filler effect) and/or as nucleation sites during the formation and development of the geopolymeric network. The effects of these structural modifications will be further evaluated through mechanical property analysis and water absorption tests, in order to correlate the evolution of porosity with the macroscopic behavior of the produced materials.

### 3.3. Differential Scanning Calorimetry (DSC) Analysis

Differential Scanning Calorimetry (DSC) analysis was performed to investigate the thermal behavior of the geopolymer samples and to identify possible changes in the physicochemical transformations occurring within the matrix. The DSC curves provide information on the thermal events associated with dehydration, structural rearrangements, and possible modifications induced by graphene incorporation. Figure 11 shows the DSC curves of the geopolymer samples (Geop 2, Geop 4, Geop 5, and Geop 6) recorded over the temperature range from room temperature to 500 °C.

The thermal behavior of the investigated systems, evaluated by differential scanning calorimetry (DSC), shows an overall comparable response among the different formulations within the temperature range from room temperature to 500 °C. All samples exhibit a main endothermic event located approximately between 130 and 140 °C, which can be mainly attributed to the removal of physically adsorbed water and water molecules retained within the porous structure of the geopolymeric matrix. This phenomenon is commonly associated with the release of weakly bound water molecules located within pores and aluminosilicate gel domains. The comparison of the DSC curves shows that the reference formulation Geop 2 exhibits a relatively more intense endothermic peak compared with the graphene-modified samples. Conversely, the graphene-containing composites (Geop 4, Geop 5, and Geop 6) display a progressive decrease in peak intensity. This behavior may be related to a different distribution of water within the porous network and to a possible modification of the accessible pore structure induced by graphene incorporation, consistent with the variations observed through BET textural analysis. Above 200 °C, all formulations exhibit a relatively stable thermal profile, without additional thermal events associated with relevant structural transformations. In particular, the samples containing higher graphene concentrations (Geop 5 and Geop 6) show a more regular baseline trend, indicating an overall stable thermal response of the composite systems.

Overall, DSC results suggest that graphene incorporation does not negatively affect the thermal stability of the geopolymeric matrix and may influence the thermal response of the material through modifications in water distribution and microstructural organization.

### 3.4. Effect of Graphene Addition on the Water Absorption Behavior of Geopolymer Samples

The water absorption test was performed on geopolymer samples produced from waste volcanic ash activated with a 6 M alkaline solution and a liquid-to-solid ratio of 0.3 (Geop-2), which was identified during preliminary tests as the optimal ratio in terms of workability and mixture homogeneity. Subsequently, three composite samples were prepared by incorporating graphene nanoparticles during the geopolymerization process, with graphene contents of 0.1 wt.% (Geop 4), 0.2 wt.% (Geop 5), and 0.5 wt.% (Geop 6), respectively. The Geop 2 sample was selected as the reference geopolymer without graphene addition. The above-described geopolymer samples were immersed in water and maintained for 24 h according to the procedure described in the Materials and Methods section, as shown in Figure 12. During the test, the surrounding environmental conditions were monitored using a thermohygrometric sensor, which recorded the air temperature and relative humidity.

Table 5 reports the initial masses of the samples, the masses after vacuum drying, the masses after 24 h water immersion, and the corresponding water absorption values.

The preliminary vacuum drying process resulted in a slight decrease in the initial mass of all samples, which can be attributed to the removal of residual moisture retained within the porous structure of the geopolymeric matrix. After 24 h of immersion in distilled water, all formulations exhibited a mass increase, confirming the presence of water-accessible porosity within the materials. As reported in Figure 13, the reference sample Geop 2 showed the lowest water absorption value (8.19%). The incorporation of graphene nanoplatelets resulted in a moderate increase in water absorption for the Geop 4 sample containing 0.1 wt% graphene, reaching a value of 10.29%. With increasing graphene content, the Geop 5 and Geop 6 samples exhibited a progressive reduction in water absorption (9.96% and 9.58%, respectively), although the values remained slightly higher than those of the unmodified geopolymer matrix. The observed variations may be related to the influence of graphene incorporation on the microstructural organization of the geopolymer matrix. At low graphene concentrations, the presence of nanoplatelets may locally affect the matrix arrangement and generate interfacial regions capable of influencing water penetration. Conversely, at higher graphene contents, the increased amount of graphene may promote a partial pore-filling effect, contributing to a reduction in pore connectivity and limiting water uptake.

Overall, the differences observed among the investigated formulations are relatively limited (approximately 2 percentage points), indicating that graphene addition at the investigated concentrations only moderately affects the water absorption behavior of the geopolymeric matrix.

### 3.5. Comparative Evaluation of Mechanical Performance

To evaluate the effect of graphene nanoparticle incorporation on the mechanical properties of the geopolymeric system, the reference matrix Geop 2 (0 wt.% GH) and the nano-modified composites Geop 4 (0.1 wt.% GH), Geop 5 (0.2 wt.% GH), and Geop 6 (0.5 wt.% GH) were subjected to comparative fracture resistance tests using a durometer. This investigation aims to establish a correlation between the previously discussed microstructural and thermal features (XRD and DSC analyses) and the macroscopic mechanical response of the materials. The systematic comparison between the unmodified geopolymer matrix and graphene-containing formulations allows the evaluation of the influence of graphene presence and dosage on the structural cohesion of the aluminosilicate gel network. In particular, the analysis provides insights into the possible role of graphene nanoparticles in enhancing load transfer mechanisms, promoting matrix densification, and improving the overall consolidation of the solid geopolymeric structure. The average failure loads obtained from the comparative mechanical tests are reported in Table 6.

The mechanical response of the geopolymer specimens shows an increase in the maximum failure load with increasing graphene nanoparticle content. The reference geopolymer (Geop 2), prepared without graphene, exhibited the lowest load-bearing capacity, with an average failure load of approximately 0.150 kN. The incorporation of 0.1 wt.% graphene (Geop 4) increased the maximum load to approximately 0.184 kN, corresponding to an increase of about 23% with respect to the reference material. A further increase in graphene content to 0.2 wt.% (Geop 5) produced an average failure load of 0.209 kN, corresponding to an improvement of approximately 39% compared with Geop 2. The highest mechanical performance was obtained for Geop 6, containing 0.5 wt.% graphene, which reached an average failure load of approximately 0.290 kN. This value represents an increase of approximately 94% relative to the reference geopolymer. The progressive increase in failure load suggests that graphene nanoparticles effectively reinforce the geopolymer matrix. This behavior can be attributed to several mechanisms, including improved stress transfer between the aluminosilicate matrix and the graphene nanoparticles, crack-bridging effects, and inhibition of crack propagation. Moreover, graphene may contribute to a more compact microstructure by reducing the development of structural defects and microvoids, resulting in enhanced mechanical resistance. Although these measurements cannot be directly considered standardized compressive strength values because of the reduced specimen dimensions and the instrumental limitations, the results provide comparative evidence that graphene addition improves the mechanical performance of the investigated geopolymer system (Figure 14).

### 3.6. Characterization and Performance Evaluation of Luminescent Resin-Coated Geopolymer Samples

To complete the microstructural, thermal, and mechanical characterization of the geopolymeric substrate, the final stage of this study was devoted to the functional evaluation of the hybrid system obtained through the deposition of an epoxy coating modified with photoluminescent powder. The resin synthesis protocol and coating application procedure were previously described in detail in the Materials and Methods section; therefore, the present section focuses on the evaluation of the optical properties of the developed system. The photoluminescent behavior of the coated geopolymer samples was initially investigated through direct exposure to natural sunlight, according to the previously defined experimental protocol. Each sample was subjected to solar irradiation for 15 min to promote the excitation of the luminescent particles incorporated into the epoxy resin matrix. During the activation stage, the specimens were placed on a reflective aluminum foil support, which was used to maximize the amount of incident radiation and enhance the irradiation of the lateral surfaces of the coated geopolymer prototypes. Figure 15 shows the experimental configuration adopted and the visual appearance of the samples during solar exposure.

All photographs were taken under identical camera settings, ensuring a consistent qualitative and comparative evaluation of luminescence. After the irradiation period, the samples were removed from the light source and visually monitored under low-light conditions to evaluate the persistence of the emitted luminescence. The photoluminescent response was monitored through photographic acquisition at predefined time intervals, with each image associated with the corresponding luminescence decay time. The results of the time-dependent monitoring are reported in Figure 16, where the images collected at different time intervals after removal of the light source are presented for each sample. This analysis allows a qualitative comparison of the duration of the luminescent effect and the evaluation of the influence of the coating formulation on the energy storage and light emission capability of the developed system.

The photoluminescent behavior of the epoxy-coated geopolymer specimens (Geop 2, Geop 4, Geop 5, and Geop 6) was qualitatively evaluated by visual observation in a completely dark environment following 15 min of natural solar irradiation. Figure 16 illustrates the temporal evolution of the visible afterglow from the end of the excitation period (t = 0 min) up to t = 17 min.

Initial emission and general decay (t = 0–5 min): Immediately after solar activation (t = 0 min), all specimens exhibited an intense visible emission, characterized by the typical cyan–blue color of the commercial photoluminescent phosphor. During the first minutes of observation, all formulations showed the expected rapid decrease in emission intensity, which is characteristic of persistent luminescent materials and is associated with the progressive release of trapped charge carriers responsible for the afterglow phenomenon.

Comparison among the developed formulations (t = 7–17 min): Although the initial emission appeared qualitatively similar for all specimens, differences became progressively evident during the decay stage. The reference geopolymer (Geop 2) and the formulation containing the lowest graphene content (Geop 4) exhibited a visibly reduced afterglow after approximately 10 min and were no longer distinguishable by visual inspection after about 12 min. In contrast, the formulations containing higher graphene contents maintained a detectable residual emission for longer periods. In particular, Geop 5 remained visually observable up to approximately 12 min, whereas Geop 6 exhibited the longest visible persistence, remaining distinguishable until approximately 17 min under the adopted experimental conditions.

Since the present investigation was based exclusively on qualitative visual observations, the mechanisms responsible for the improved persistence cannot be definitively established. Nevertheless, the results suggest that the incorporation of graphene into the geopolymer matrix may indirectly influence the performance of the photoluminescent coating. One possible explanation is related to modifications of the microstructural characteristics of the geopolymer substrate. As indicated by the SEM, BET, and mechanical analyses, graphene incorporation influenced the organization of the geopolymeric matrix, which may have provided a more uniform and mechanically stable support for the epoxy-phosphor coating. A more homogeneous substrate could promote a more regular coating thickness and a more uniform distribution of the photoluminescent particles, potentially contributing to a longer visually detectable afterglow. In addition, considering the intrinsically high thermal conductivity of graphene, it may be hypothesized that the presence of graphene within the geopolymer matrix could influence local heat transfer during solar activation. Such an effect, if confirmed by dedicated investigations, might contribute to preserving the photoluminescent performance of the phosphor by limiting localized thermal effects. However, this hypothesis cannot be confirmed within the scope of the present study and should be regarded as a possible interpretation requiring further experimental validation.

Overall, the qualitative observations demonstrate the feasibility of combining recycled volcanic ash-based geopolymers, graphene nanoplatelets, and photoluminescent coatings into a single multifunctional composite material. Although this work represents a preliminary proof-of-concept, the longer afterglow visually observed for the formulation containing 0.5 wt% graphene highlights the potential of this approach for the future development of sustainable multifunctional materials intended for passive nighttime visibility, safety marking, and orientation systems in civil infrastructures. Future investigations based on quantitative photometric measurements, accelerated durability tests, and optimization of both the geopolymer substrate and the photoluminescent coating will be necessary to fully assess the practical potential of the proposed system.

## 4. Conclusions

This study investigated the feasibility of developing sustainable multifunctional geopolymer composites by valorizing recycled volcanic ash from Mount Etna as an aluminosilicate precursor combined with graphene nanoplatelets and a photoluminescent epoxy coating. The first stage of the investigation was devoted to optimizing the liquid-to-solid (L/S) ratio in order to identify the most suitable geopolymer matrix. The combined results obtained from SEM, EDS, BET, PSD, and XRD analyses indicated that the formulation with L/S = 0.3 (Geop 2) provided the most balanced overall characteristics among the investigated mixtures. This formulation exhibited a homogeneous microstructure, an appropriate pore distribution, and mineralogical features consistent with a successful geopolymerization process while preserving the crystalline phases naturally present in the volcanic ash. Based on these findings, Geop 2 was selected as the reference matrix for the subsequent incorporation of graphene nanoplatelets.

Graphene was subsequently incorporated at concentrations of 0.1, 0.2, and 0.5 wt% to evaluate its influence on the optimized geopolymer matrix. The combined characterization by SEM, EDS, BET, PSD, XRD, and DSC indicated that graphene addition did not produce detectable alterations to the geopolymeric network or compromise the thermal stability of the material within the investigated temperature range. Instead, the presence of the nano-additive was associated with moderate changes in the textural properties of the composites, accompanied by a progressive reduction in specific surface area and pore volume as the graphene content increased. Comparative mechanical tests, performed under identical experimental conditions, suggested an improvement in the mechanical response of the graphene-modified formulations with respect to the reference geopolymer, while water absorption measurements showed only limited variations among the investigated compositions, indicating that graphene incorporation did not substantially modify the overall water uptake behavior of the matrix.

The optimized geopolymers were subsequently employed as substrates for a transparent epoxy coating containing commercial photoluminescent phosphors. After 15 min of natural solar irradiation, all formulations exhibited visible persistent luminescence under dark conditions. Among the investigated specimens, the composite containing 0.5 wt% graphene (Geop 6) showed the longest visually detectable afterglow under the adopted experimental conditions. Although the present evaluation was based exclusively on qualitative visual observations, the results suggest that the characteristics of the graphene-modified geopolymer substrate may contribute to the persistence of the photoluminescent emission and support the feasibility of developing sunlight-activated photoluminescent geopolymer composites.

Overall, the proposed approach demonstrates a simple and relatively low-cost strategy for converting abundant volcanic waste into multifunctional materials with reduced environmental impact. The integration of recycled volcanic ash, graphene nanoplatelets, and photoluminescent technology provides a promising platform for the development of sustainable composites with potential applications in passive nighttime visibility, road and pedestrian safety, emergency guidance systems, architectural elements, and smart infrastructure where external power sources are undesirable or unavailable.

Finally, it should be emphasized that the present work represents a preliminary laboratory-scale proof-of-concept study. Further investigations involving quantitative photometric characterization, long-term durability assessment, environmental aging, standardized mechanical testing, and optimization of both the geopolymer matrix and the photoluminescent coating will be necessary to fully evaluate the performance of the proposed material. Nevertheless, the encouraging results obtained suggest that volcanic ash-based geopolymer composites modified with graphene and combined with photoluminescent coatings represent a promising starting point for the future development of sustainable and intelligent multifunctional construction materials.

## Figures and Tables

**Figure 1:**
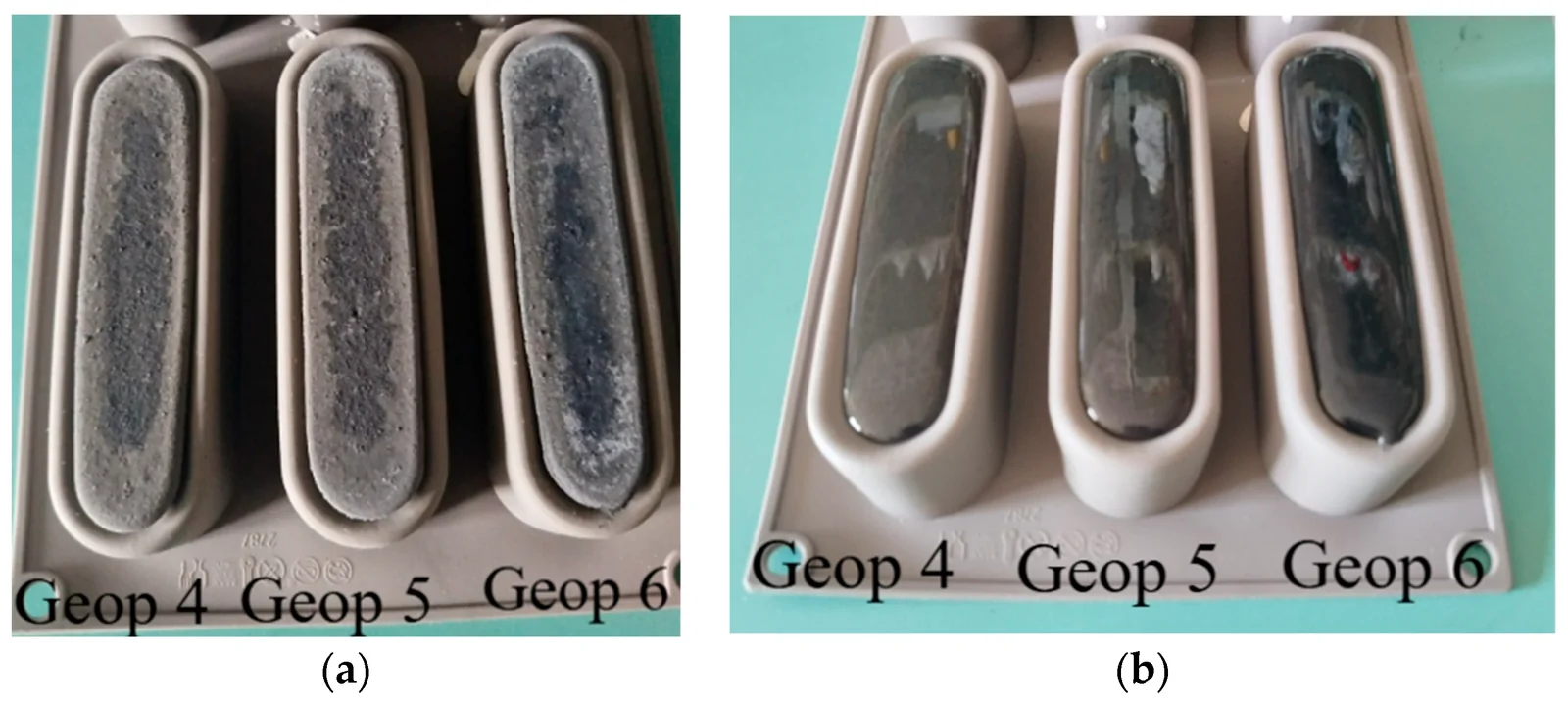
(**a**) Geopolymer mixtures cast into silicone molds; (**b**) geopolymer specimens after thermal curing (110 °C, 24 h) and demolding.

**Figure 2:**
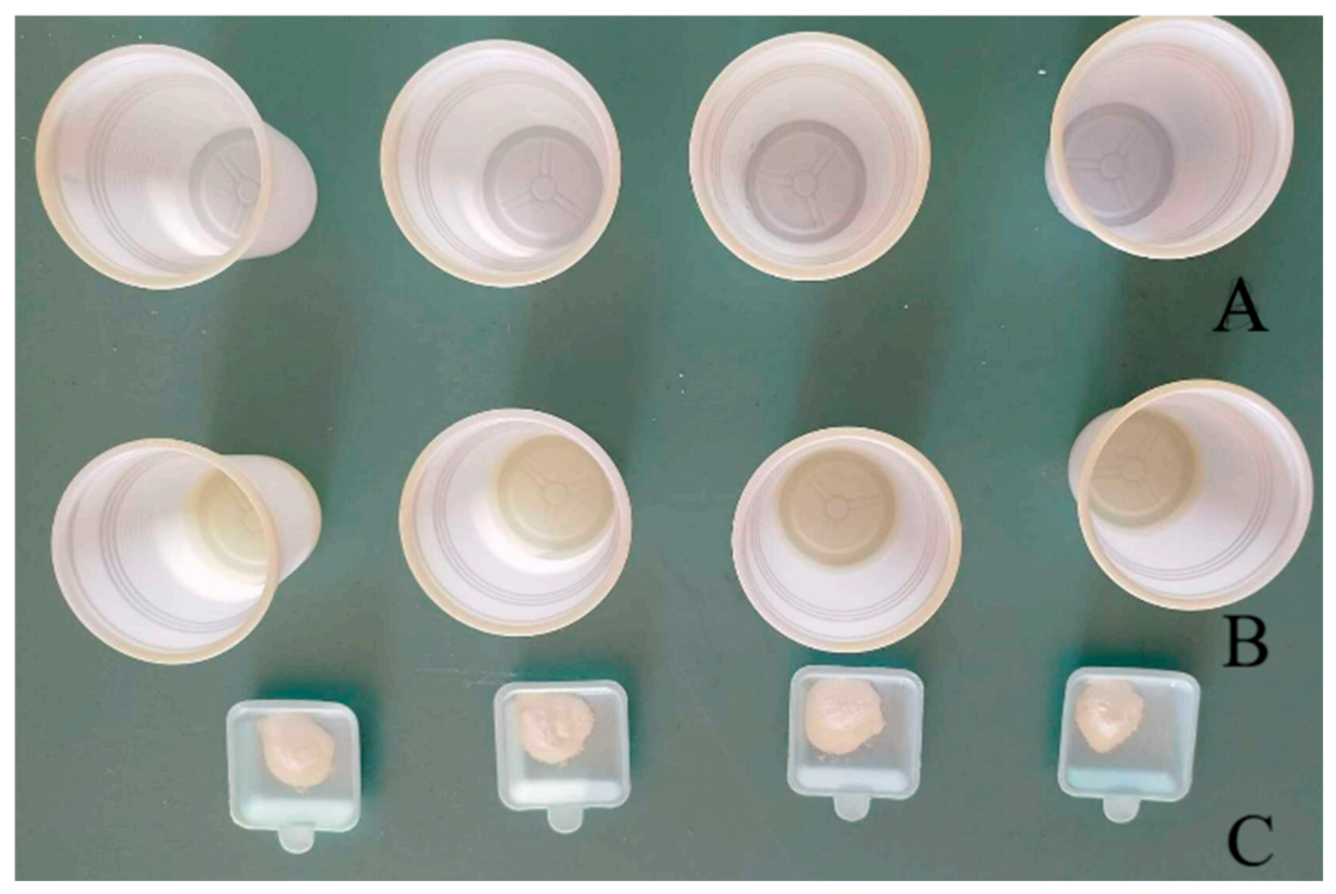
Materials used for the preparation of the photoluminescent coating: (**A**) transparent epoxy resin, Component A; (**B**) hardener, Component B; (**C**) commercial photoluminescent powder.

**Figure 3:**
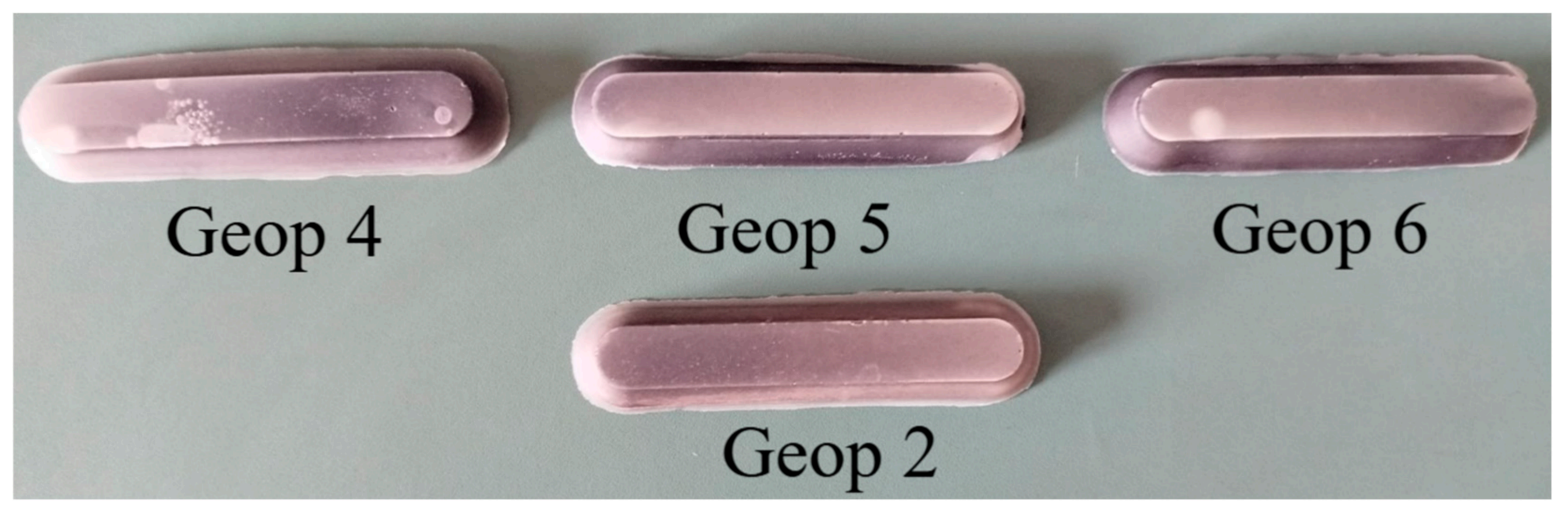
Photoluminescent geopolymer prototypes after epoxy coating curing and demolding from the silicone molds.

**Figure 4:**
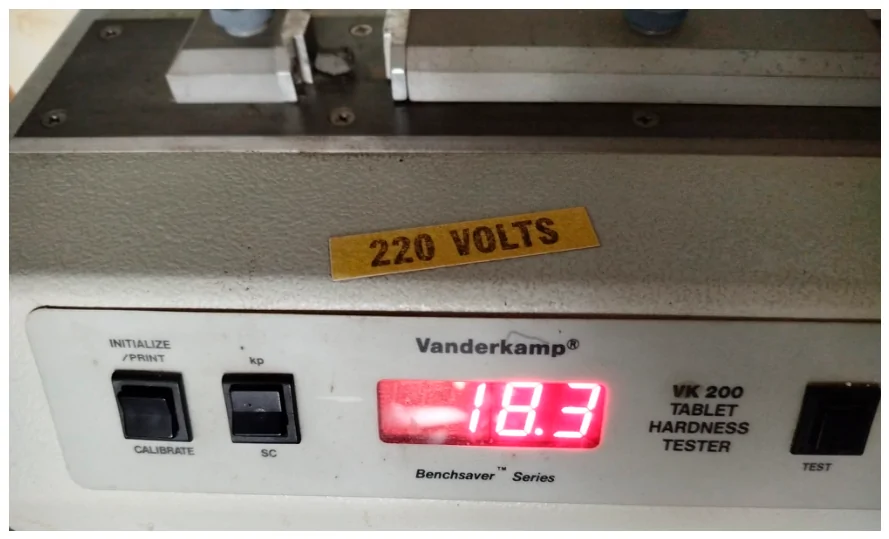
Digital durometer (VanderKamp VK200) used for the preliminary comparative mechanical evaluation of geopolymer specimens.

**Figure 5:**
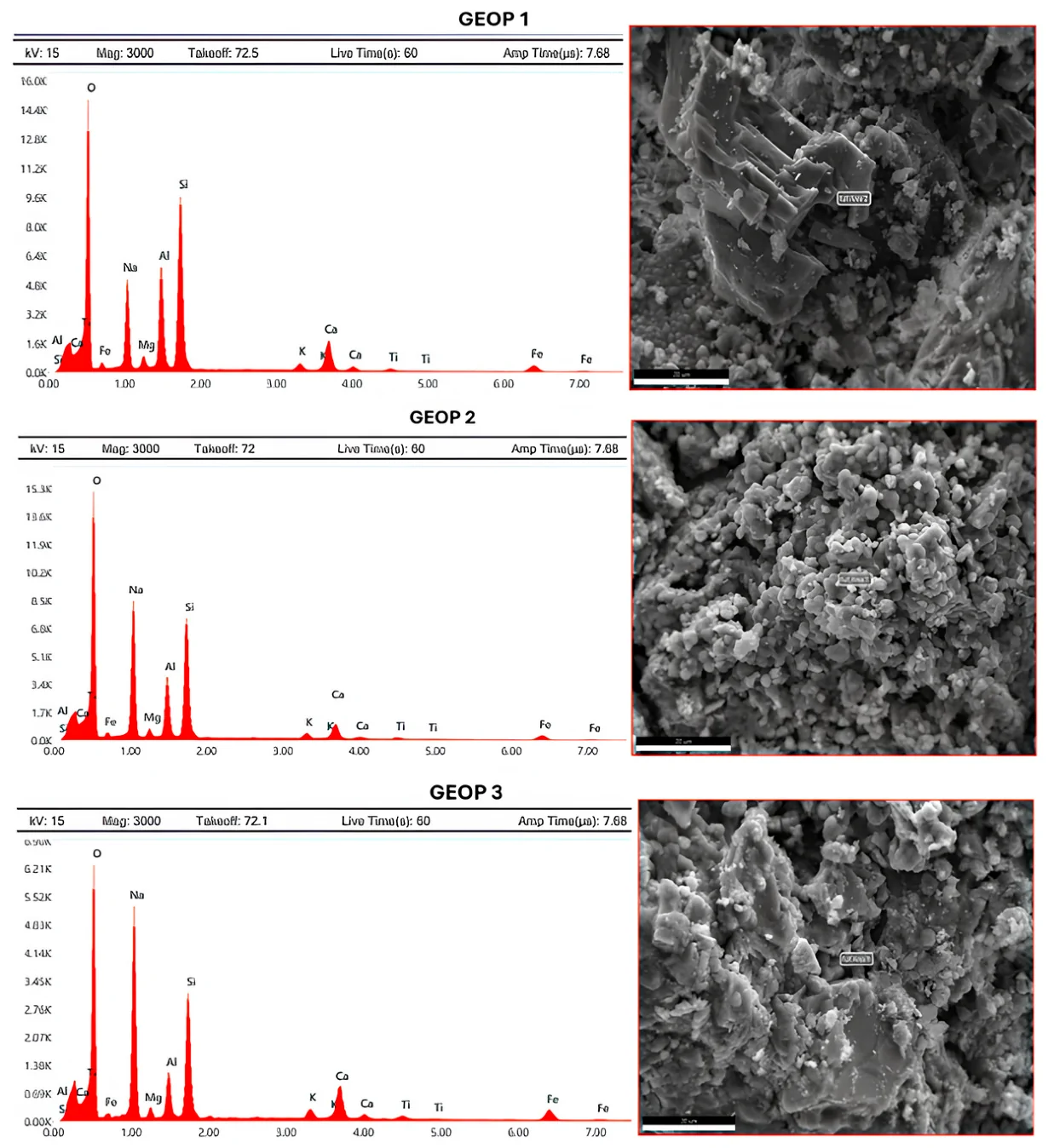
SEM micrographs and EDS analyses of geopolymer samples with different liquid-to-solid (L/S) ratios.

**Figure 6:**
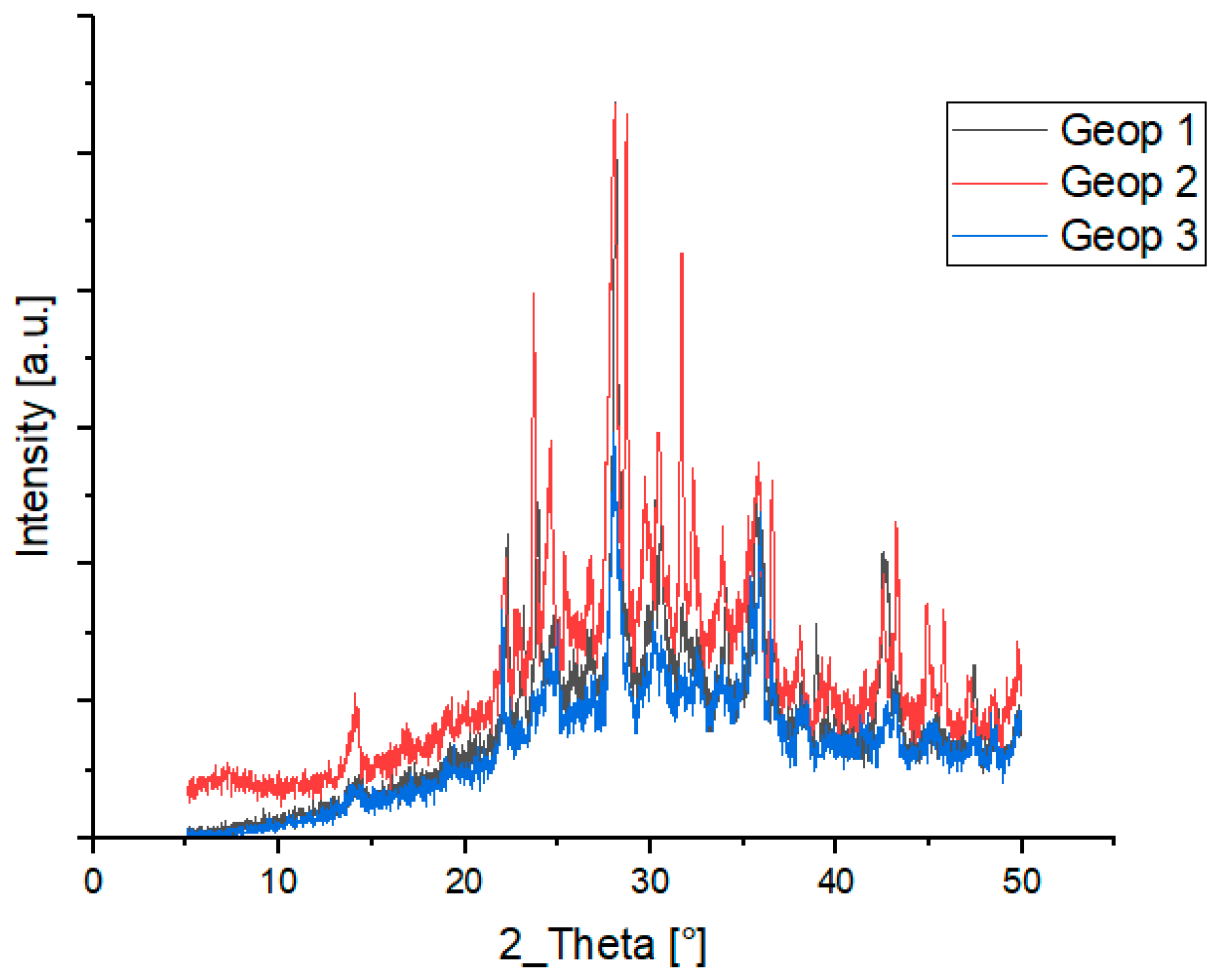
Comparative XRD patterns of Etna volcanic ash-based geopolymers synthesized at different liquid-to-solid ratios: Geop 1, Geop 2, and Geop 3. The broad amorphous halo associated with the aluminosilicate-rich geopolymeric matrix (2θ ≈ 20–38°) and the main crystalline reflections compatible with residual mineral phases, mainly plagioclase- and pyroxene-related phases, are indicated.

**Figure 7:**
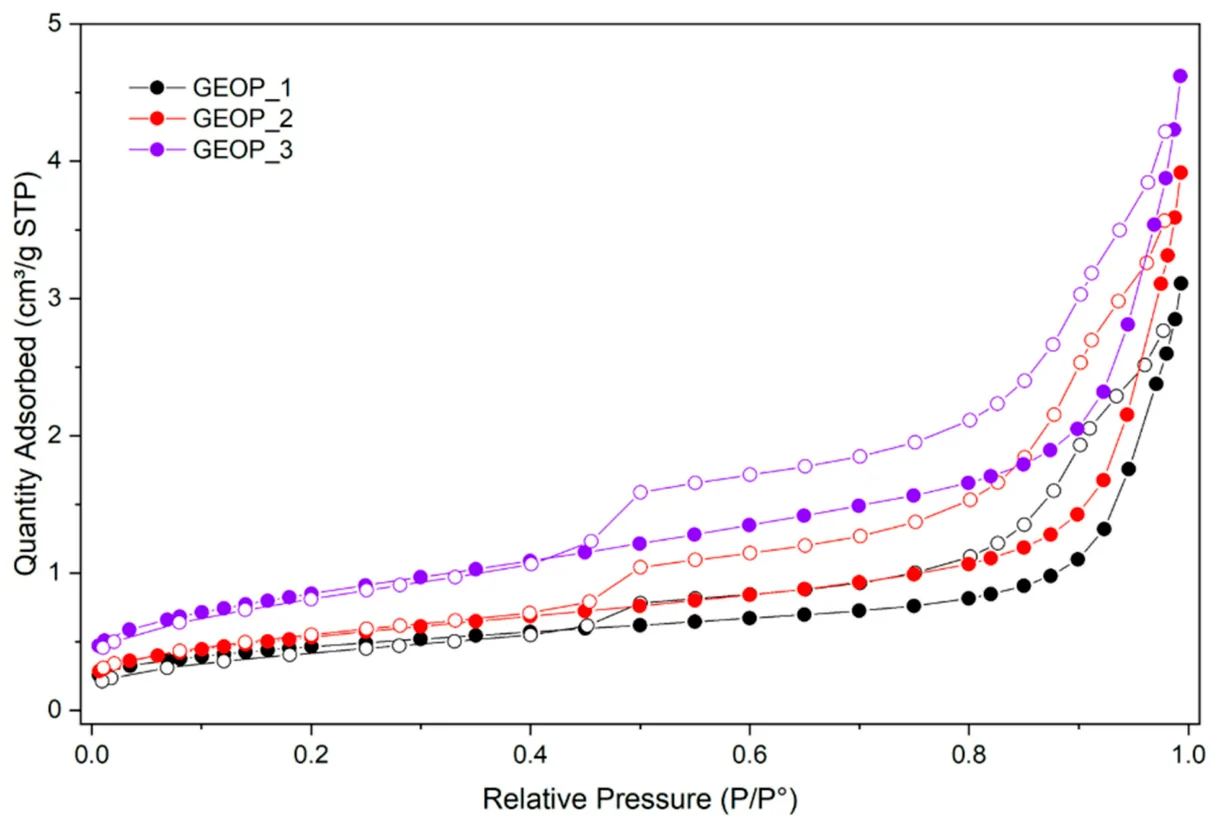
Nitrogen adsorption–desorption isotherms at 77 K for the geopolymer samples Geop 1, Geop 2, and Geop 3. Filled symbols represent the adsorption branch, whereas open symbols indicate the desorption branch.

**Figure 8:**
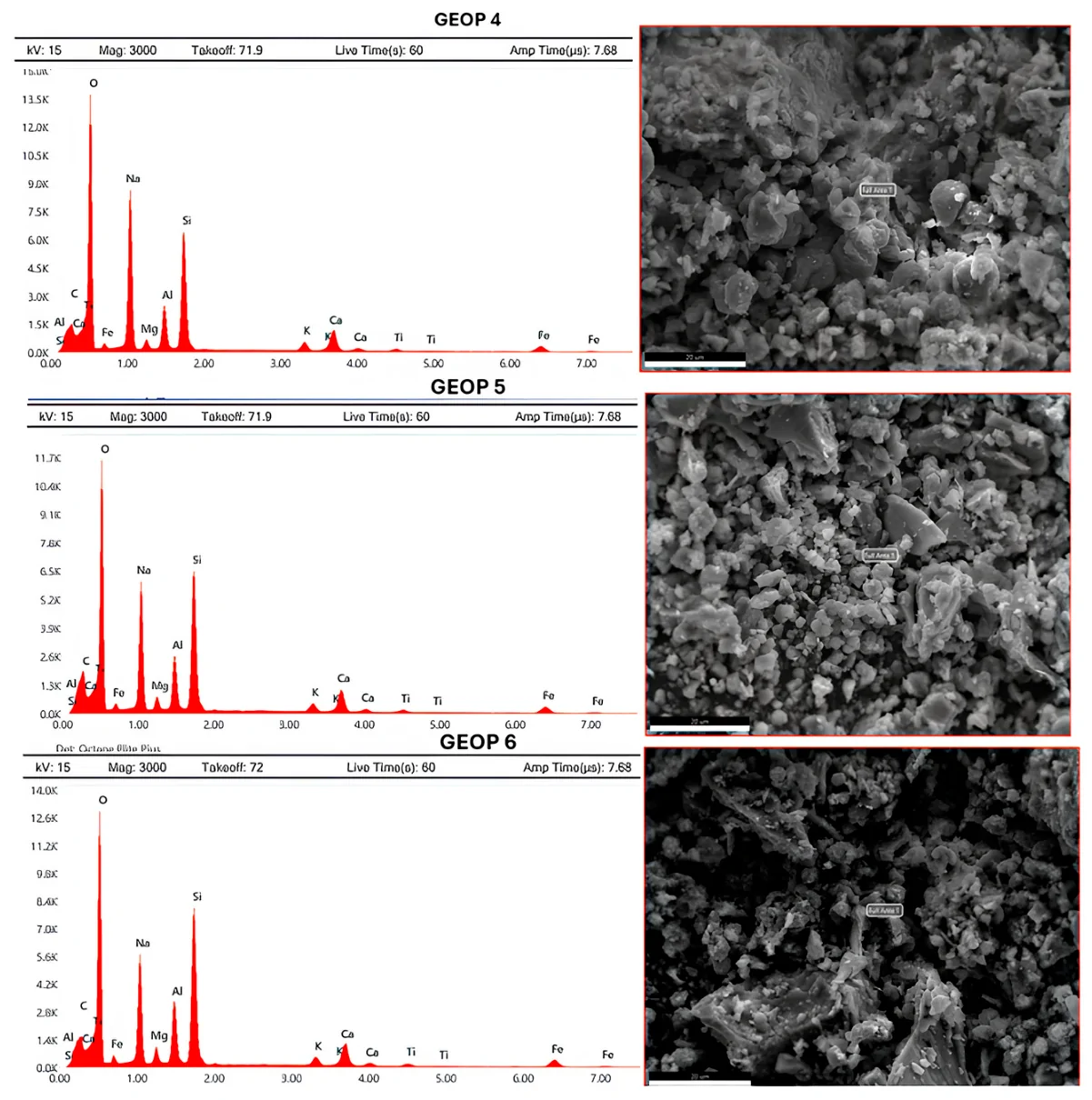
SEM images and EDS microanalyses of Geop 4, Geop 5, and Geop 6 samples prepared with a liquid-to-solid (L/S) ratio of 0.3 and different graphene contents, namely 0.1, 0.2 and 0.5 wt%, respectively.

**Figure 9:**
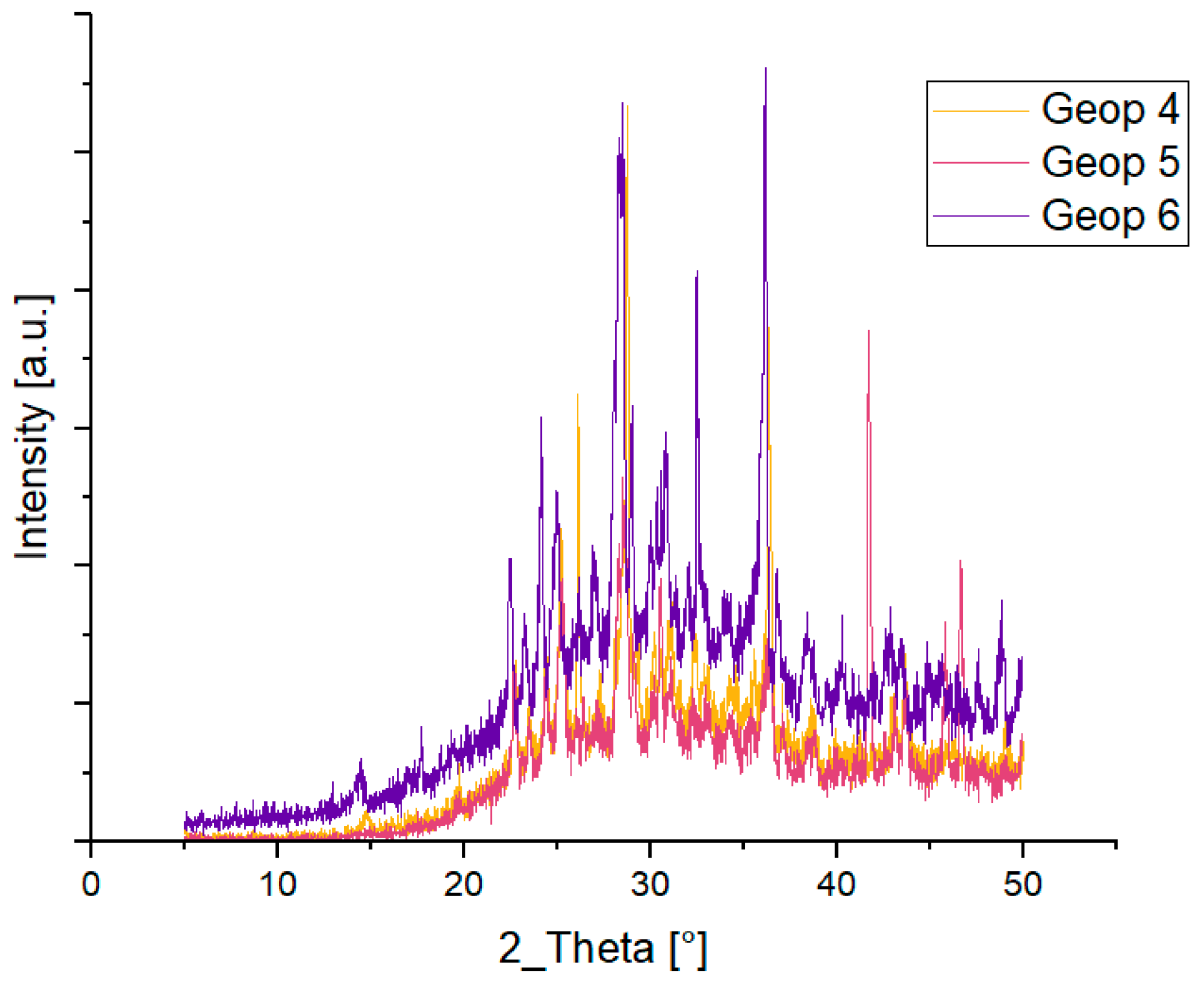
Shows the overlapped XRD patterns of Etna lava-based geopolymer samples prepared using the optimal liquid-to-solid ratio (L/S = 0.3) and containing increasing amounts of graphene nanoparticles (GH), namely 0.1 wt% (Geop 4), 0.2 wt% (Geop 5), and 0.5 wt% (Geop 6).

**Figure 10:**
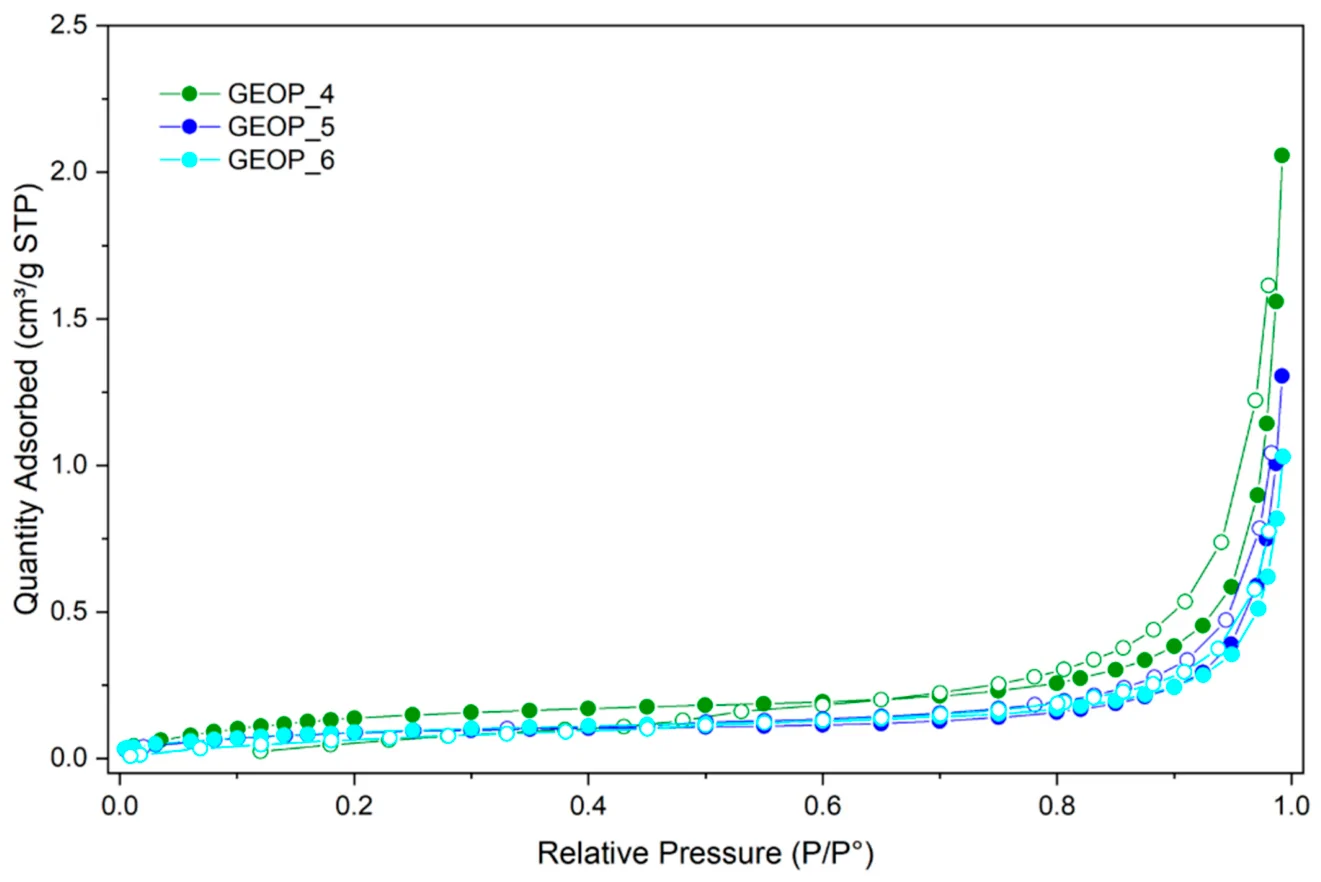
Nitrogen adsorption–desorption isotherms at 77 K for the geopolymer samples GEOP_4, GEOP_5, and GEOP_6. Filled symbols represent the adsorption branch, whereas open symbols indicate the desorption branch.

**Figure 11:**
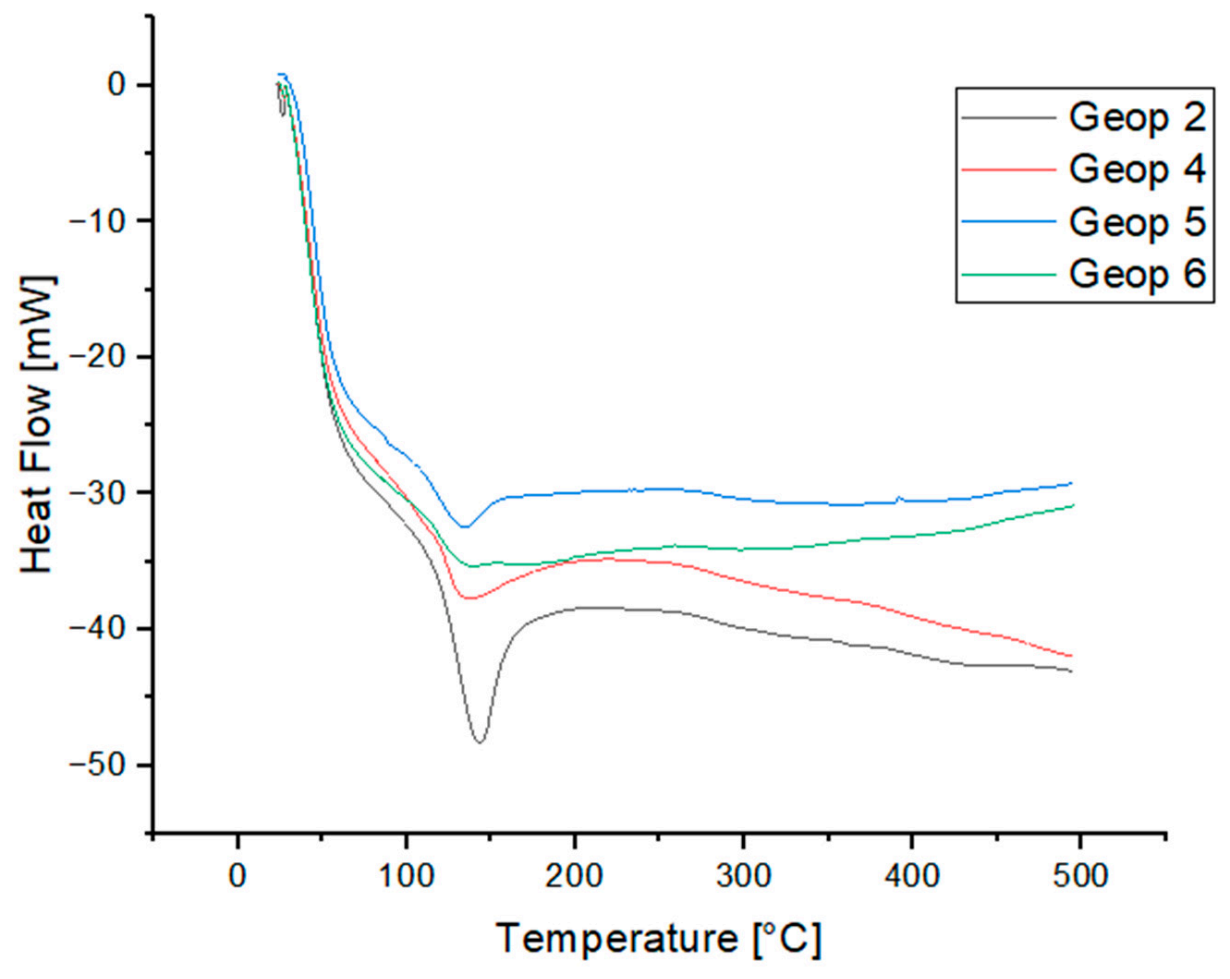
Comparative DSC curves of the reference geopolymer matrix (Geop 2) and graphene nanoparticle-reinforced composites at different weight fractions: Geop 4 (0.1 wt.%), Geop 5 (0.2 wt.%), and Geop 6 (0.5 wt.%). Endothermic processes are directed downwards.

**Figure 12:**
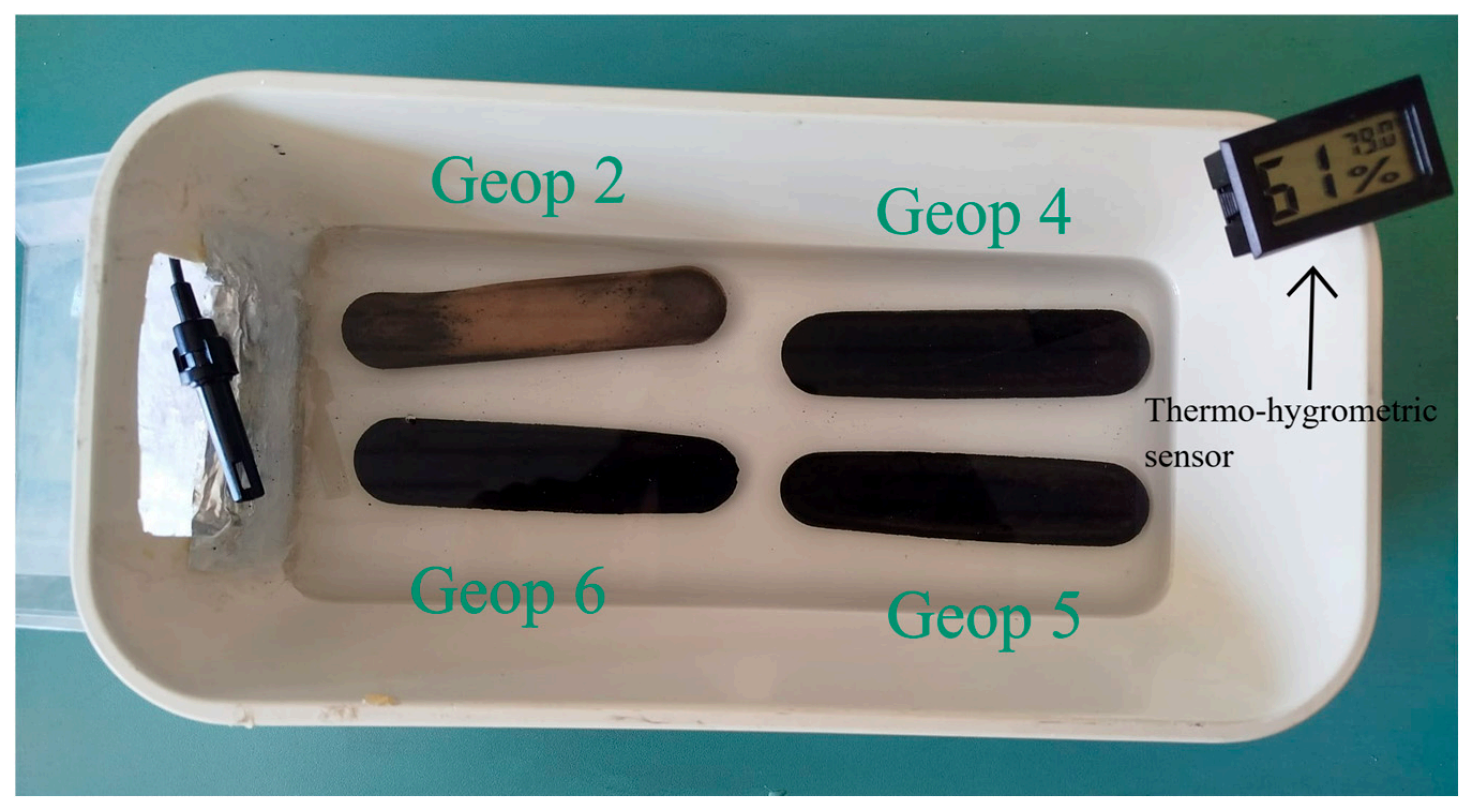
Geopolymer specimens described above immersed in water for 24 h according to the protocol reported in the Materials and Methods section. Ambient conditions were monitored using a temperature and relative humidity sensor.

**Figure 13:**
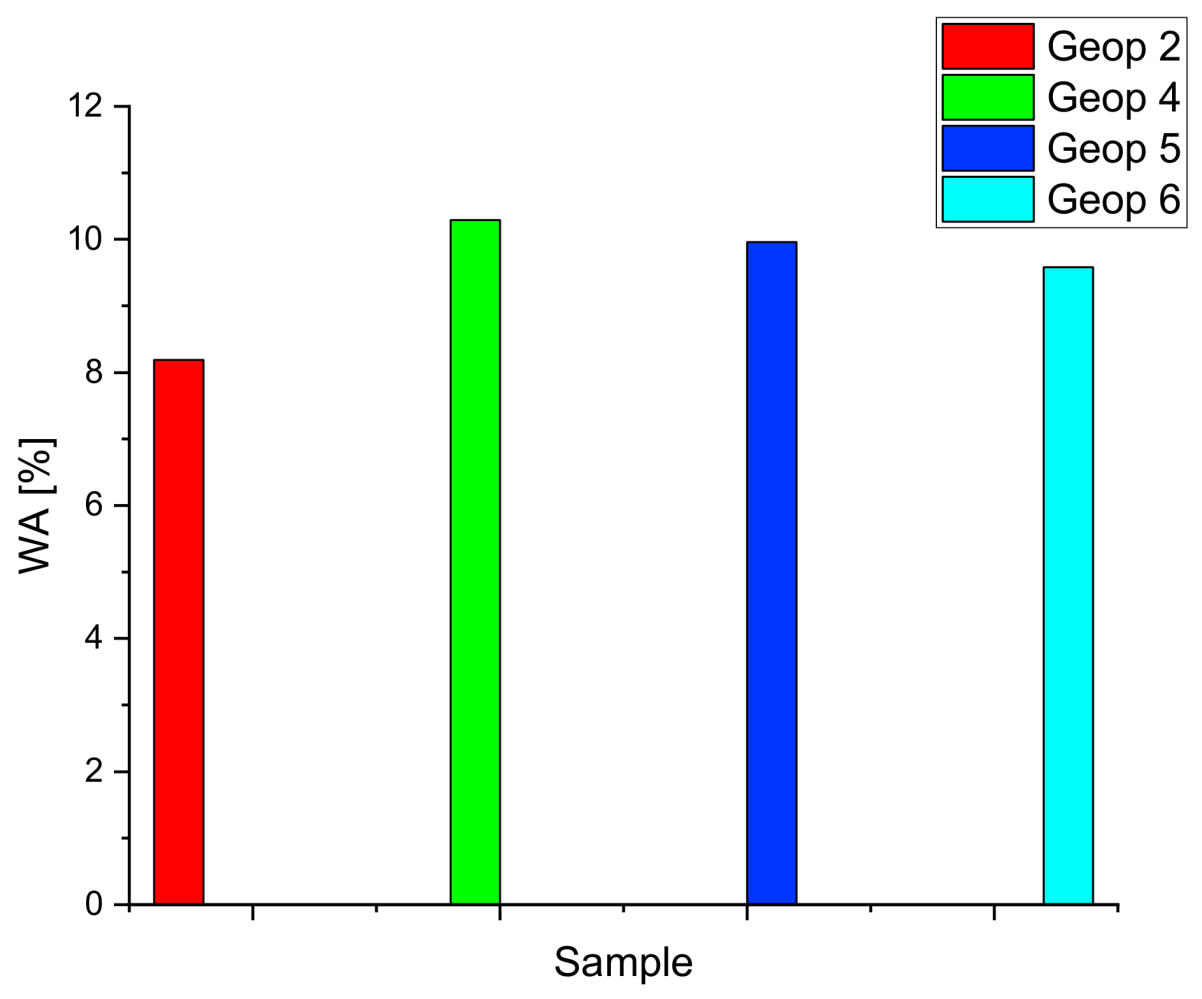
Water absorption of geopolymer composites after 24 h immersion.

**Figure 14:**
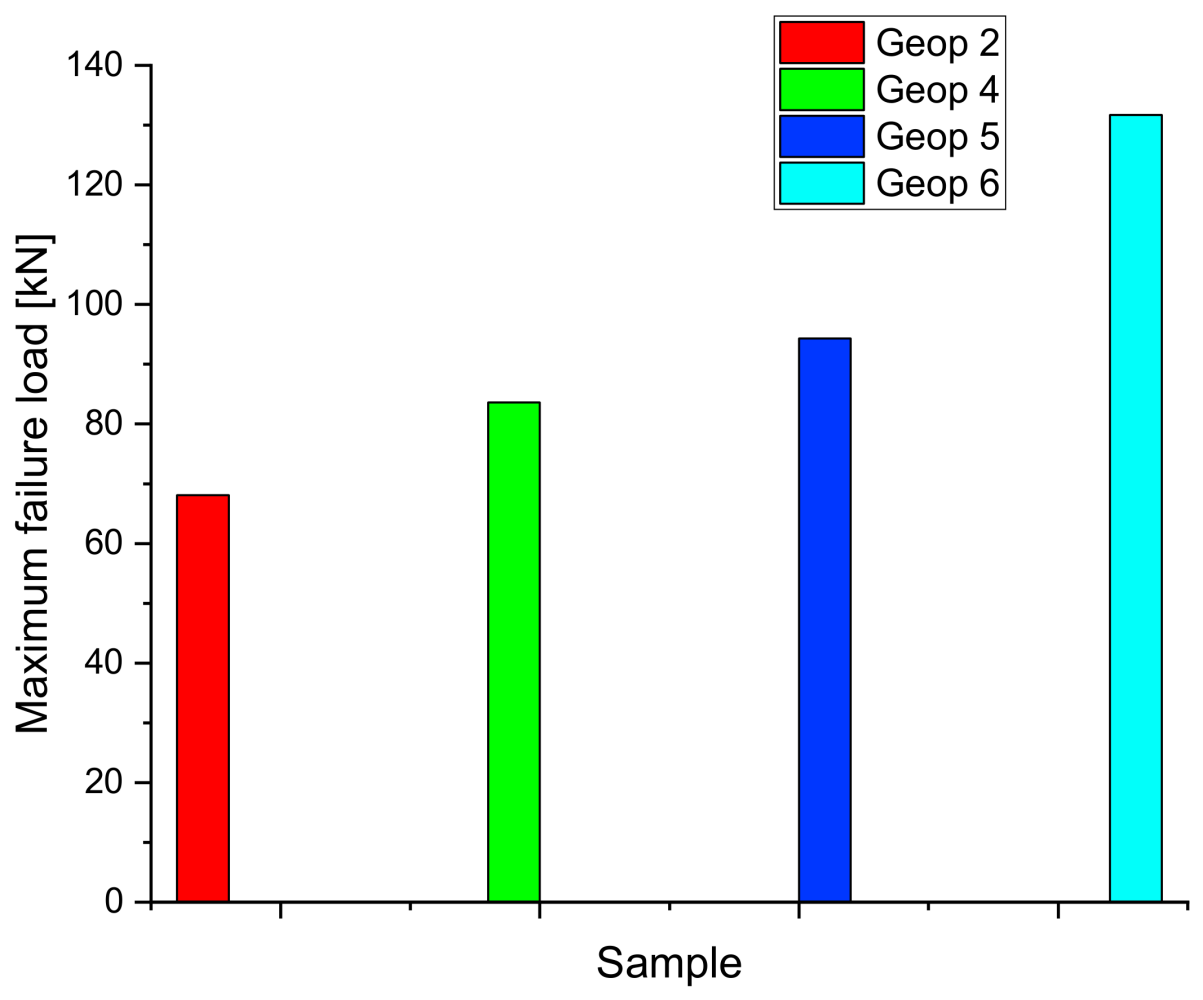
Maximum failure load of volcanic ash-based geopolymer composites (Geop 2, Geop 4, Geop 5, and Geop 6) as a function of graphene content (0, 0.1, 0.2, and 0.5 wt.%), measured using the VanderKamp VK 200 hardness tester.

**Figure 15:**
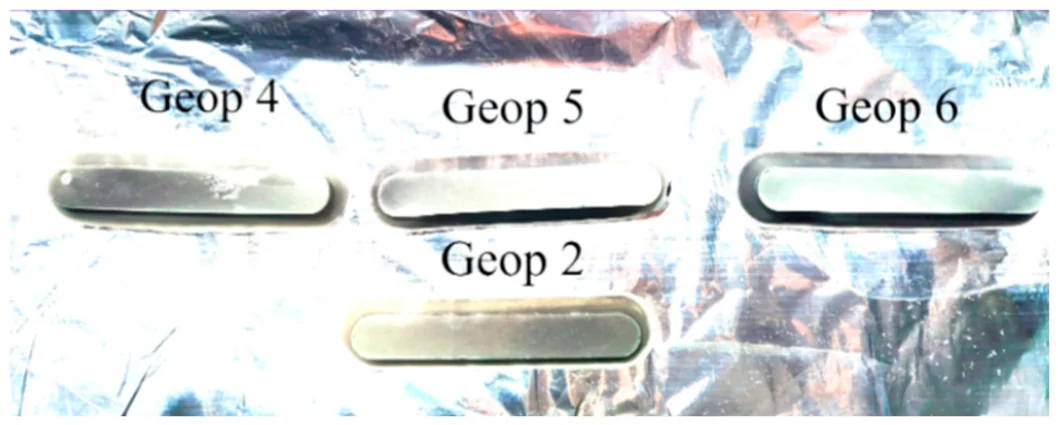
Experimental setup of the coated geopolymer samples during solar irradiation, showing the reflective aluminum support.

**Figure 16:**
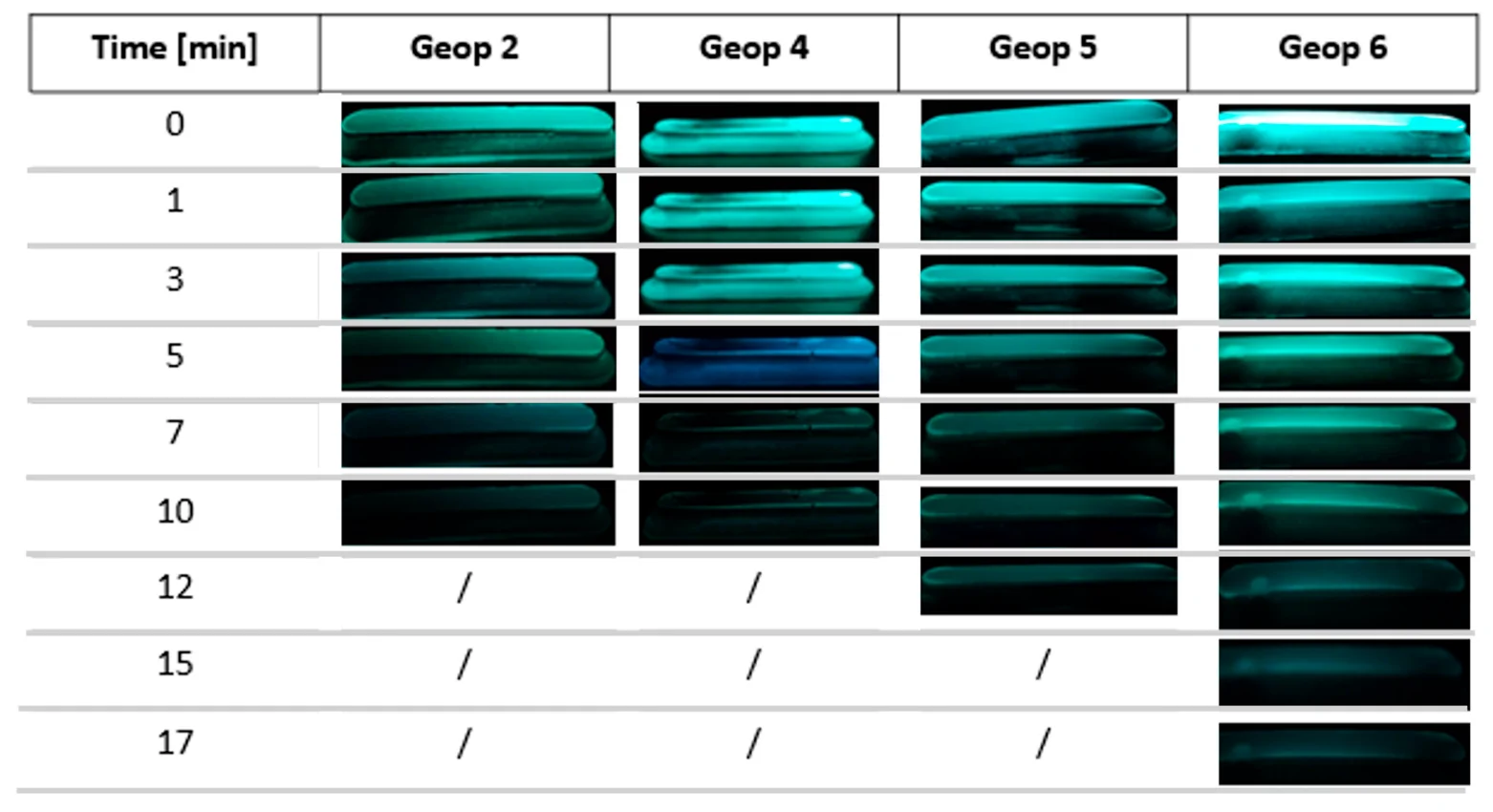
Time-dependent photographic monitoring of the photoluminescent emission of coated geopolymer samples after solar irradiation.

**Table 1:** Mix design and composition of the geopolymer samples.

Sample ID	Liquid-Solid Ratio [g/g]	Volcanic Powder [g] ± 0.01	6 M NaOH [g] ± 0.01	Graphene Np [g] ± 0.001	Volcanic Powder [wt%]	6 M NaOH [wt%]	Graphene wt [%]
**Geop 1**	0.2	30	6	0	83.33	16.67	0.00
**Geop 2**	0.3	30	10	0	75.00	25.00	0.00
**Geop 3**	0.4	30	13	0	69.77	30.23	0.00
**Geop 4**	0.3	30	10	0.030	74.94	24.98	0.09
**Geop 5**	0.3	30	10	0.060	74.89	24.96	0.19
**Geop 6**	0.3	30	10	0.150	74.72	24.91	0.49

**Table 2:** Environmental conditions recorded during the natural solar irradiation of the geopolymer specimens.

Environmental Parameter	Value	Unit
Sky conditions	Clear sky	–
Air temperature	34.8	°C
Relative humidity	49	%
Global solar irradiance	980	W·m^−2^
Atmospheric pressure	1017	h·Pa

Note: Environmental data were obtained from publicly available meteorological databases corresponding to the location and time of the solar activation test.

**Table 3:** Textural Properties of analyzed samples without graphene.

Sample	Total Pore Volume [cm^3^/g]	Specific Surface Area [m^2^/g]
Geop 1	0.00479	1.648 ± 0.013
Geop 2	0.00618	1.902 ± 0.014
Geop 3	0.00705	3.020 ± 0.024

**Table 4:** Textural Properties of analyzed samples with graphene.

Sample	Total Pore Volume [cm^3^/g]	Specific Surface Area [m^2^/g]
GEOP_4	0.00319	0.526 ± 0.015
GEOP_5	0.00132	0.317 ± 0.006
GEOP_6	0.00157	0.324 ± 0.006

**Table 5:** Mass measurements before and after vacuum drying and water absorption values of the geopolymer specimens after 24 h immersion. All reported values represent the meaning of three replicate measurements, and the corresponding standard deviations are included.

Sample	Initial Mass [g]	Dry Mass [g]	Saturated Mass [g]	Water Absorption [%]
Geop 2	28.028 ± 0.012	27.933 ± 0.010	30.220 ± 0.015	8.19 ± 0.12
Geop 4	29.056 ± 0.015	28.975 ± 0.011	31.955 ± 0.018	10.29 ± 0.15
Geop 5	30.652 ± 0.014	30.557 ± 0.012	33.600 ± 0.017	9.96 ± 0.14
Geop 6	31.713 ± 0.015	30.625 ± 0.012	33.560 ± 0.018	9.58 ± 0.14

**Table 6:** Average maximum failure load measured by the VanderKamp VK200 tester for the investigated geopolymer formulations (*n* = 3). All reported values represent the mean of three replicate measurements, and the corresponding standard deviations are included.

Sample ID	Load [kp]	Load [N]	Load [kN]	Increase [%]
Geop 2	15.3 ± 0.4	150.03 ± 3.9	0.150 ± 0.004	—
Geop 4	18.8 ± 0.5	184.35 ± 4.6	0.184 ± 0.005	+23
Geop 5	21.3 ± 0.6	208.88 ± 5.9	0.209 ± 0.006	+39
Geop 6	29.6 ± 0.8	290.32 ± 7.8	0.290 ± 0.008	+94

## Data Availability

The original contributions presented in this study are included in the article. Further inquiries can be directed to the corresponding authors.

## References

[1] Omar A.S., Gupta R., Bijanu A., Arya R., Mishra D., Singh A., Salammal S.T. (2022). Progress in Fabrication and Manufacturing of Sodium Aluminosilicate Materials (Geopolymers) as Protective Coating Materials: A Review. J. Polym. Res..

[2] Ricciotti L., Lucariello D., Perrotta V., Apicella A., Aversa R. (2025). Sustainable Alkali-Activated and Geopolymer Materials: What Is the Future for Italy?. Recycling.

[3] Rogers J.J., MacKenzie K.J.D., Trompetter W.J. (2017). Aluminosilicate Inorganic Polymers (Geopolymers) Containing Rare Earth Ions: A New Class of Photoluminescent Materials. J. Mater. Sci..

[4] Manu B.A. (2024). Innovative Construction Materials: Advancing Sustainability, Durability, Efficiency, and Cost-Effectiveness in Modern Infrastructure. Int. J. Res. Publ. Rev..

[5] Tahwia A.M., Aldulaimi D.S., Abdellatief M., Youssf O. (2024). Physical, Mechanical and Durability Properties of Eco-Friendly Engineered Geopolymer Composites. Infrastructures.

[6] Ong S.-W., Heah C.-Y., Liew Y.-M., Abdullah M.M.A.B., Ho L.-N., Pakawanit P., Khalid M.S., Wan Muhammad W.H.B., Ooi W.-E., Hang Y.-J. (2023). Green development of fly ash geopolymer via casting and pressing approaches: Strength, morphology, efflorescence and ecological properties. Constr. Build. Mater..

[7] Madeo L., Macario A., Candamano S., De Luca P. (2025). Recovery of Vegetable Fibers from Licorice Processing Waste and a Case Study for Their Use in Green Building Products. Clean. Technol..

[8] Hassan A., Arif M., Shariq M., Alomayri T., Pereira S. (2023). Fire resistance characteristics of geopolymer concrete for environmental sustainability: A review of thermal, mechanical and microstructure properties. Environ. Dev. Sustain..

[9] Provis J.L. (2018). Alkali-Activated Materials. Cem. Concr. Res..

[10] Duxson P., Lukey G.C., van Deventer J.S.J. (2006). Evolution of Gel Structure during Thermal Processing of Na-Geopolymer Gels. Langmuir.

[11] Zhang M., Xu H., Phalé Zeze A.L., Zhang J. (2023). Metakaolin-Based Geopolymer Composites Modified by Epoxy Resin and Silane: Mechanical Properties and Organic–Inorganic Interaction Mechanism. Appl. Clay Sci..

[12] Caputo P., Calandra P., Loise V., Le Pera A., Putz A.-M., Abe A.A., Madeo L., Teltayev B., Luprano M.L., Alfè M. (2022). When Physical Chemistry Meets Circular Economy to Solve Environmental Issues: How the ReScA Project Aims at Using Waste Pyrolysis Products to Improve and Rejuvenate Bitumens. Sustainability.

[13] Madeo L., Macario A., Napoli F., De Luca P. (2026). Licorice (*Glycyrrhiza glabra*): Botanical Aspects, Multisectoral Applications, and Valorization of Industrial Waste for the Recovery of Natural Fiber in a Circular Economy Perspective. Fibers.

[14] Rathod N., Chippagiri R., Ralegaonkar R.V. (2023). Cleaner Production of Geopolymer Materials: A Critical Review of Waste-Derived Activators. Mater. Today Proc..

[15] Djobo J.N.Y., Elimbi A., Tchakouté H.K., Kumar S. (2017). Volcanic ash-based geopolymer cements/concretes: The current state of the art and perspectives. Environ. Sci. Pollut. Res..

[16] Luhar S., Rajamane N.P., Corbu O., Luhar I. (2019). Impact of Incorporation of Volcanic Ash on Geopolymerization of Eco-Friendly Geopolymer Composites: A Review. IOP Conf. Ser. Mater. Sci. Eng..

[17] Scanferla P., Finocchiaro C., Gharzouni A., Barone G., Mazzoleni P., Rossignol S. (2023). High Temperature Behavior of Sodium and Potassium Volcanic Ashes-Based Alkali-Activated Materials (Mt. Etna, Italy). Constr. Build. Mater..

[18] Gatta G.D., Comboni D. Volcanic Raw Materials for the Low Environmental Impact Production of Pozzolanic Mortars. In Atti del Convegno/Conference Proceedings; 2022. https://hdl.handle.net/2434/913601.

[19] Occhipinti R., Portale S., Lanzafame G., Gimeno D., Kudrna Prašek M., Mazzoleni P., Barone G. (2024). Alkali Activated Materials from Tajogaite Volcanic Ash (La Palma, Spain): A Green Recovery after the 2021 Eruption. Volcanica.

[20] Matsimbe J., Dinka M., Olukanni D., Musonda I. (2022). Geopolymer: A Systematic Review of Methodologies. Materials.

[21] Singh S.K., Badkul A., Pal B. (2024). Sustainable next-generation single-component geopolymer binders: A review of mechano-chemical behaviour and life-cycle cost analysis. J. Mater. Cycles Waste Manag..

[22] Irum S., Shabbir F., Salahuddin H., Waqas R.M. (2025). Performance evaluation of sustainable recycled aggregates geopolymer concrete using cement and graphite nano/micro platelets under heat and ambient curing. Emergent Mater..

[23] Wang W., Zhong Z., Kang X., Ma X. (2023). Physico-Mechanical Properties and Micromorphological Characteristics of Graphene Oxide Reinforced Geopolymer Foam Concrete. J. Build. Eng..

[24] Zhang Y.J., He P.Y., Zhang Y.X., Chen H. (2018). A Novel Electroconductive Graphene/Fly Ash-Based Geopolymer Composite and Its Photocatalytic Performance. Chem. Eng. J..

[25] Madeo L., Macario A., Sapia P., De Luca P. (2025). Synergistic Integration of Graphene Nanoparticles in Colloidal TiO_2_ for Grätzel Cells (DSSC). J. Compos. Sci..

[26] Yin L., Xie D., Gong K., Shi C., Qian X., Zhou K. (2023). Phase Change Materials Encapsulated in Coral-Inspired Organic–Inorganic Aerogels for Flame-Retardant and Thermal Energy Storage. ACS Appl. Nano Mater..

[27] Madeo L., Macario A., De Luca P. (2025). Eco-Sustainable Photoluminescent Epoxy Composites Incorporating Etna Volcanic Ash as a Natural Waste for Decorative Building Applications. Procedia Environ. Sci. Eng. Manag..

[28] Singla R., Senna M., Mishra T., Alex T.C., Kumar S. (2022). High Strength Metakaolin/Epoxy Hybrid Geopolymers: Synthesis, Characterization and Mechanical Properties. Appl. Clay Sci..

[29] Fu K., Tian Y., Zhu Z., Fa S., Zhang Q. (2023). Highly-Transparent Fluorinated Epoxy Coating Prepared via Ring-Opening of Thiolactone and Thiol-Click Reactions for Robust Omniphobicity. Prog. Org. Coat..

[30] Zheng X., Lei M., Yang M., Wu H., Chen J., Lin F., Wang D., Zheng G., Xu Y., Lin Q. (2026). Dual-Functional Waterborne Coatings Featuring Enhanced Anticorrosion and Persistent Luminescence Enabled by Rare-Earth Doping. Prog. Org. Coat..

[31] Wu J., Zhou X., Zhang D., Li L., Jiang S., Xiang G., Wang Y., Tang X., Li J., Cao Z. (2023). Luminescent Properties of Lanthanide Complexes and Applications in Security Anti-Counterfeiting and Information Encryption Based on pH Response. J. Lumin..

[32] Zheng B., Fan J., Chen B., Qin X., Wang J., Wang F., Deng R., Liu X. (2022). Rare-Earth Doping in Nanostructured Inorganic Materials. Chem. Rev..

[33] Manna A.S., Ghosh S., Ghosh T., Karchaudhuri N., Das S., Roy A., Maiti D.K. (2025). Smart Luminescent Materials for Emerging Sensors: Fundamentals and Advances. Chem.–Asian J..

[34] Elsawy H., Sedky A., Abou Taleb M.F., El-Newehy M.H. (2022). Colour-Switchable and Photoluminescent Polyvinyl Chloride for Multifunctional Smart Applications. Luminescence.

[35] Abdollahi A., Rahmanidoust M., Hanaei N., Dashti A. (2023). All-in-One Photoluminescent Janus Nanoparticles for Smart Technologies: Organic Light-Emitting Diodes, Anticounterfeiting, and Optical Sensors. Eur. Polym. J..

[36] Chen D., Chen Y., Zhu Z., Luo F., Wu F., Zhou Q., Guo C. (2024). Smart Window with Reversible and Instantaneous Photoluminescence Based on Microsphere Structure. ACS Appl. Mater. Interfaces.

[37] Ricciotti L., Frettoloso C., Franchino R., Pisacane N., Aversa R. (2025). Geopolymer Materials: Cutting-Edge Solutions for Sustainable Design Building. Sustainability.

[38] Lo Bianco A., Armetta F., Calvino M.M., Gagliardo Briuccia G.E., Macalik B., Hreniak D., Saladino M.L., Lazzara G., Cavallaro G. (2026). Invisible Near-Infrared Luminescent Marker Incorporating Egyptian Blue in Halloysite-Based Geopolymer Mortars: Applications in Covert Tagging. J. Alloys Compd..

[39] Rathinavel N., Murugesan A., Jaisankar K., Abdul Hameed M.J.S.A. (2025). Innovative Materials for Next-Generation Defence Applications: Implications for Civil Infrastructure in Cost, Performance, and Mass Production. Innovative Materials for Next-Generation Defense Applications: Cost, Performance, and Mass Production.

[40] Dorrian M. (2021). Peter Wilson and Mark Dorrian in Conversation. J. Archit..

[41] Rouquerol J., Llewellyn P., Rouquerol F., Llewellyn P.L., Rodriguez-Reinoso F., Rouquerol J., Seaton N. (2007). Is the BET Equation Applicable to Microporous Adsorbents?. Characterization of Porous Solids VII, Proceedings of the 7th International Symposium on the Characterization of Porous Solids (COPS-VII), Aix-en-Provence, France, 26–28 May 2005.

[42] Barrett E.P., Joyner L.G., Halenda P.P. (1951). The Determination of Pore Volume and Area Distributions in Porous Substances. I. Computations from Nitrogen Isotherms. J. Am. Chem. Soc..

[43] Harkins W.D., Jura G. (1944). Surfaces of Solids. XIII. A Vapor Adsorption Method for the Determination of the Area of a Solid without the Assumption of a Molecular Area, and the Areas Occupied by Nitrogen and Other Molecules on the Surface of a Solid. J. Am. Chem. Soc..

